# Biochemical and Expression Analyses of the Rice Cinnamoyl-CoA Reductase Gene Family

**DOI:** 10.3389/fpls.2017.02099

**Published:** 2017-12-12

**Authors:** Hye Lin Park, Seong Hee Bhoo, Mi Kwon, Sang-Won Lee, Man-Ho Cho

**Affiliations:** ^1^Graduate School of Biotechnology and College of Life Sciences, Kyung Hee University, Yongin, South Korea; ^2^Institute of Biological Chemistry, Washington State University, Pullman, WA, United States

**Keywords:** rice, cinnamoyl-CoA reductase, monolignol pathway, lignin, biotic/abiotic stress

## Abstract

Cinnamoyl-CoA reductase (CCR) is the first committed enzyme in the monolignol pathway for lignin biosynthesis and catalyzes the conversion of hydroxycinnamoyl-CoAs into hydroxycinnamaldehydes. In the rice genome, 33 genes are annotated as *CCR* and *CCR-like* genes, collectively called *OsCCR*s. To elucidate the functions of *OsCCR*s, their phylogenetic relationships, expression patterns at the transcription levels and biochemical characteristics were thoroughly analyzed. Of the 33 *OsCCR*s, 24 of them encoded polypeptides of lengths similar to those of previously identified plant CCRs. The other nine OsCCRs had much shorter peptide lengths. Phylogenetic tree and sequence similarities suggested OsCCR4, 5, 17, 18, 19, 20, and 21 as likely candidates for functional CCRs in rice. To elucidate biochemical functions, OsCCR1, 5, 17, 19, 20, 21, and 26 were heterologously expressed in *Escherichia coli* and the resulting recombinant OsCCRs were purified to apparent homogeneity. Activity assays of the recombinant OsCCRs with hydroxycinnamoyl-CoAs revealed that OsCCR17, 19, 20, and 21 were biochemically active CCRs, in which the NAD(P)-binding and NADP-specificity motifs as well as the CCR signature motif were fully conserved. The kinetic parameters of enzyme reactions revealed that feruloyl-CoA, a precursor for the guaiacyl (G)-unit of lignin, is the most preferred substrate of OsCCR20 and 21. This result is consistent with a high content (about 70%) of G-units in rice lignins. Phylogenetic analysis revealed that OsCCR19 and 20 were grouped with other plant CCRs involved in developmental lignification, whereas OsCCR17 and 21 were closely related to stress-responsible CCRs identified from other plant species. In agreement with the phylogenetic analysis, expression analysis demonstrated that *OsCCR20* was constitutively expressed throughout the developmental stages of rice, showing particularly high expression levels in actively lignifying tissues, such as roots and stems. These results suggest that *OsCCR20* is primarily involved in developmental deposition of lignins in secondary cell walls. As expected, the expressions of *OsCCR17* and *21* were induced in response to biotic and abiotic stresses, such as *Magnaporthe grisea* and *Xanthomonas oryzae* pv. *oryzae* (*Xoo*) infections, UV-irradiation and high salinity, suggesting that these genes play a role in defense-related processes in rice.

## Introduction

Plants are constantly confronted by both biotic and abiotic stresses, leading to significant reductions in their productivity (Strange and Scott, 2005; Vinocur and Altman, 2005; Oerke, 2006; Chakraborty and Newton, 2011). Abiotic stresses, including drought, salinity, and extreme temperature, are the primary factors in crop loss and can reduce the average yields of major crop plants by more than 50% (Boyer, 1982; Oerke, 2006). Biotic stresses, such as infection by pathogens, can cause serious reduction of cereal production (Strange and Scott, 2005; Oerke, 2006; Chakraborty and Newton, 2011). It has been reported that actual losses of worldwide rice production due to biotic stresses, in the period of 2001–2003, comprised an estimated 37.4% of the total attainable production (Oerke, 2006). To cope with biotic and abiotic stresses, plants have developed a wide array of defense mechanisms such as the fortification of cell walls, production of phytoalexins, and accumulation of reactive oxygen species (Moura et al., 2010; Ahuja et al., 2012; Großkinsky et al., 2012; Miedes et al., 2014; Rejeb et al., 2014).

Lignin is complex aromatic polymer primarily composed of *p*-hydroxyphenyl (H)-, G- and syringyl (S)-units derived from monolignols. Lignin is predominantly deposited in the secondary cell walls of xylem and fiber cells and makes the cell walls rigid and impervious (Campbell and Sederoff, 1996; Donaldson, 2001; Bonawitz and Chapple, 2010; Vanholme et al., 2010). The lignified secondary cell walls are important for the water conduction and mechanical support of vascular plants, and serve as a physical barrier against pathogens and herbivores (Campbell and Sederoff, 1996; Donaldson, 2001; Vanholme et al., 2010; Miedes et al., 2014). In addition to developmental deposition, the synthesis and deposition of lignin-related phenolics are induced in response to biotic and abiotic stresses (Moura et al., 2010; Hamann, 2012; Miedes et al., 2014).

The biosynthetic pathway of lignin is divided into two branches: the general phenylpropanoid pathway from phenylalanine to hydroxycinnamoyl-CoAs, and the monolignol pathway from hydroxycinnamoyl-CoAs to monolignols. These monolignols include *p*-coumaroyl, coniferyl, and sinapyl alcohols (Davin et al., 2008; Vanholme et al., 2010; Miedes et al., 2014). In addition to lignin biosynthesis, the phenylpropanoid pathway is used in the synthesis of a vast array of phenolic compounds including phytoalexins, phenylpropanoid conjugates and flavonoids (Dixon et al., 2002; Ahuja et al., 2012; Großkinsky et al., 2012; Cho and Lee, 2015). Hydroxycinnamoyl-CoA esters are subsequently channeled into the lignin branch pathway to produce monolignols through hydroxycinnamaldehydes via two reductive steps catalyzed by CCR and cinnamyl alcohol dehydrogenase (Nimz et al., 1975; Gross, 1981; Lüderitz and Grisebach, 1981; Lacombe et al., 1997). In addition to serving as intermediates in lignin biosynthesis, hydroxycinnamaldehydes, and monolignols can play a role as defensive compounds and act as precursors for lignan biosynthesis (Keen and Littlefield, 1979; Barber et al., 2000; Davin et al., 2008; König et al., 2014; Satake et al., 2015; Teponno et al., 2016). In rice, the expression of phenylpropanoid pathway genes were induced in response to biotic and abiotic stresses, such as *M. grisea* infection and UV-irradiation, and led to the synthesis of phenolic phytoalexins (Ishihara et al., 2008; Park et al., 2013, 2014; Cho and Lee, 2015). It has been also reported that hydroxycinnamic acid amides were synthesized and deposited in the cell walls of *Bipolaris oryzae* infected tissues, suggesting that these amides were involved in physical defense against the pathogen (Ishihara et al., 2008, 2011).

CCR is the first enzyme of the monolignol pathway, and catalyzes the conversion of *p*-coumaroyl-, feruloyl-, and sinapoyl-CoAs to *p*-coumaraldehyde, coniferaldehyde, and sinapaldehyde, respectively (Gross, 1981; Davin et al., 2008; Vanholme et al., 2010). Homologs of *CCR* gene families have been reported to be diverse in plant species, including 11 genes in *Arabidopsis thaliana*, nine in *Populus trichocarpa*, 26 in rice (*Oryza sativa*) and 10 in *Eucalyptus grandis* (Costa et al., 2003; Kawasaki et al., 2006; Shi et al., 2010; Carocha et al., 2015). Functional studies of CCRs has been performed in some plant species including *A. thaliana, Eucalytus gunnii*, soybean (*Glycine max*), poplar (*P. euramericana*), maize (*Zea mays*), switchgrass (*Panicum virgatum*), and wheat (*Triticum aestivum*) (Lüderitz and Grisebach, 1981; Sarni et al., 1984; Goffner et al., 1994; Lacombe et al., 1997; Pichon et al., 1998; Lauvergeat et al., 2001; Goujon et al., 2003; Ma, 2007; Escamilla-Treviño et al., 2010; Tamasloukht et al., 2011). Multiple homologs of *CCR* genes have been reported to play different roles in the same plant species (Lauvergeat et al., 2001; Ma, 2007; Escamilla-Treviño et al., 2010). In *A. thaliana, AtCCR1* is involved in developmental lignification, while *AtCCR2* participates in stress and elicitor responses (Lauvergeat et al., 2001). Down-regulation of *AtCCR1* has been observed to lead the reduction of lignin contents up to 50% in *A. thaliana* (Goujon et al., 2003). Although several studies have reported defense-related functions of *CCR-like* genes in rice (Kawasaki et al., 2006; Bart et al., 2010), biochemical and physiological roles of rice *CCR* and *CCR-like* genes are largely unknown.

In the MSU Rice Genome Annotation Project (RGAP) database, we found 33 genes annotated as *CCR* and *CCR-like* genes, collectively called *OsCCR*s. The gene expression profiles of different developmental stages, organs and stress conditions, and the activity of enzyme toward hydroxycinnamoyl-CoA substrates were examined for the functional characterization of *OsCCR*s in rice. An activity assay of recombinant OsCCR proteins revealed that OsCCR17, 19, 20, and 21 were biochemically functional CCRs in rice. Expression and phylogenetic analyses were performed to elucidate the physiological role of *OsCCR*s, and suggested that *OsCCR19* and *20* are primarily involved in developmental lignification, while *OsCCR17* and *21* likely play a role in defense responses.

## Materials and methods

### Plant growth and materials

Sterilized seeds of wild-type rice plants (*O. sativa* L. spp. *Japonica* cv. *Dongjin*) were germinated on Murashige and Skoog (MS) medium (Duchefa, Harlem, Netherlands) in a growth chamber with a 12 h photoperiod and temperature of 28°C. Ten-day old seedlings were transferred to soil and grown in a greenhouse at 28°C during the day and 20°C at night. Stem and leaf samples were collected from 10-week-old rice plants, and panicle samples were collected from 14-week-old rice plants. Root and shoot samples were collected from 10-day old rice seedlings.

*p*-Coumaric acid, ferulic acid, sinapic acid, coenzyme-A (CoA) and reduced β-nicotinamide adenine dinucleotide phosphate (NADPH) for hydroxycinnamoyl-CoA production were purchased from Sigma-Aldrich (St. Louis, MO, USA). Reagents for buffers, media and other solutions were obtained from Sigma-Aldrich and Duchefa.

### Multiple sequence alignments and phylogenetic analysis of OsCCRs

Deduced protein sequences of OsCCRs and functional CCRs identified from other plant species were retrieved from the MSU RGAP database (http://rice.plantbiology.msu.edu/, Kawahara et al., 2013) and the National Center for Biotechnological Information (https://www.ncbi.nlm.nih.gov/) database, respectively. Multiple amino acid sequence alignment was performed with Clustal-W (Thompson et al., 1994), and a phylogenetic analysis was conducted with MEGA ver. 6 (Tamura et al., 2013) using the neighbor-joining method.

### Cloning of *OsCCR*s

Total RNA was isolated from 8-week-old rice leaves with RNAiso (Takara, Shiga, Japan). The first cDNA was synthesized using the total RNA and SuPrimeScript RT premix with an oligo dT primer (GeNet Bio, Daejeon, Korea). Cloning primers for *OsCCR* genes were designed according to the sequences in the MSU RGAP database. The amplification primers and polymerase chain reaction (PCR) conditions are provided in Supplementary Table 1. PCR was performed using Solg™ Pfu DNA Polymerase (SolGent, Daejeon, Korea). The resulting PCR products were subcloned into the pGEM™-T Easy vector (Promega, Madison, WI, USA) or pJET 1.2 blunt cloning vector (Thermo Scientific, Carlsbad, CA, USA). After sequence confirmation, each *OsCCR* gene was cut out with the appropriate restriction enzymes and inserted into the pET28a(+) vector (Novagen, Madison, WI, USA). The resulting *OsCCR*/pET28a(+) constructs were individually transformed into *E. coli* BL21(DE3) cells for heterologous expression of OsCCRs.

### Expression and purification of recombinant OsCCRs

The *E. coli* transformants harboring the *OsCCR*/pET28a(+) construct were grown at 37°C until an OD_600_ of ~0.6 in LB medium containing kanamycin (25 μg/mL) was achieved. At that point, 0.1 mM isopropyl β-D-thiogalactopyranoside (IPTG) was added in the culture for induction. After additional incubation at 18 or 25°C for 16 h, the cells were harvested by centrifugation (5,000 g for 15 min). Cell pellets were resuspended in phosphate-buffered saline (137 mM NaCl, 2.7 mM KCl, 10 mM Na_2_HPO_4_, 2 mM KH_2_PO_4_) supplemented with lysozyme (1 mg/mL) and phenylmethylsulfonyl fluoride (1 mM). The resuspended cells were sonicated on ice, and the crude protein extracts were obtained by centrifugation (15,900 g for 20 min, 4°C). The crude protein samples were mixed with Ni-NTA Agarose beads (Qiagen, Hilden, Germany) and incubated at 4°C for 2 h with agitation. The mixtures were packed into a chromatography column and washed three times with a five-column volume of 20 mM imidazole in Tris buffer (50 mM Tris, pH 8.0, 300 mM NaCl). The recombinant OsCCRs were eluted with 50–100 mM imidazole in Tris buffer. The eluted proteins were analyzed by sodium dodecyl sulfate-polyacrylamide gel electrophoresis (SDS-PAGE).

### Enzymatic synthesis of hydroxycinnamoyl-CoAs

For the OsCCR activity assay, hydroxycinnamoyl-CoAs were synthesized by the method described by Beuerle and Pichersky (2002). *Arabidopsis* 4-coumarate:CoA ligase 1 (*At4CL1*) was cloned from *A. thaliana* cDNA, and the resulting gene was inserted into pET28a(+) vector (Supplementary Table 1) (Stuible and Kombrink, 2001). The recombinant At4CL1 was expressed in *E. coli* and purified with Ni-NTA Agarose beads according to the methods described above. To synthesize the hydroxycinnamoyl-CoA esters, 3.3 mg hydroxycinnamic acid (*p*-coumaric, ferulic, or sinapic acids), 2 mg CoA, and 6.9 mg ATP were dissolved into a total volume of 10 mL of 50 mM Tris-HCl pH 7.5 buffer containing 2.5 mM MgCl_2_. The reaction was initiated by the addition of 0.25 mg purified At4CL1. After a 5 h incubation at room temperature with agitation, 6.9 mg ATP, 2 mg CoA, and 0.25 mg purified At4CL1 enzyme were added to the reaction mixture, and the incubation was continued at room temperature for 12 h. Ammonium acetate (0.4 g) was added to the mixture to halt the reaction. Hydroxycinnamoyl-CoA esters were purified using Sep-Pak® Vac tC_18_ cartridge (Waters, Milford, MA, USA) preconditioned with consecutive washes of MeOH, H_2_O, and 4% ammonium acetate solution (five column-volumes each). The reaction mixture was loaded on the preconditioned cartridge, and the column was rinsed with 4% ammonium acetate solution. The hydroxycinnamoyl-CoA esters were eluted with H_2_O. Fractions containing the hydroxycinnamoyl-CoA esters were identified by UV/Vis spectra recorded using a V-550 UV/Vis-spectrophotometer (Jasco, Tokyo, Japan), and the purified products were lyophilized for storage.

### CCR activity assay and determination of kinetic parameters

OsCCR activity was measured according to the methods of Lüderitz and Grisebach (1981). The reaction mixture consisted of 0.1 mM NADPH, 30 μM hydroxycinnamoyl-CoA, and 5 μg of purified recombinant OsCCR protein in 100 mM sodium/potassium phosphate buffer (pH 6.25) to a total volume of 500 μL. The enzyme reactions were carried out at 30°C. The reaction was initiated by an addition of recombinant OsCCR protein, and decreases in A_366_ were monitored for 10 min by a Cary 300 Bio UV/Vis-spectrophotometer (Varian, Mulgrave, Victoria, Australia). For determination of *K*_M_ and *V*_max_, the substrates were used at concentrations of 5–50 μM. *K*_M_ and *V*_max_ were determined by extrapolation from Lineweaver-Burk plots. The enzyme assays were carried out in quadruplicate and the result represented the mean ± standard deviation.

### UV and salt treatment

Wild-type Dongjin rice plants were grown in a greenhouse for 8 weeks after germination. UV-C treatment of rice plants were performed using the methods described by Park et al. (2013). UV-treated rice leaves were collected 1, 24, and 48 h after UV treatment.

To treat salt stress, rice seedlings were hydroponically grown on MS medium (Duchefa), and 10 day-old rice seedlings were treated with 250 mM NaCl. After 1, 3, 6, 12, and 24 h salt treatments, rice seedlings were collected for the analysis of *OsCCR* expression.

### Analysis of *OsCCR* gene expression

The public transcriptomic analysis data of *OsCCR* genes in various rice developmental stages as well as under biotic [*M. grisea, Xoo*, and *X. oryzae* pv. *oryzicolar* (*Xoc*) infections] and abiotic stresses (drought, salt and cold) were downloaded from the Genevestigator plant biology database (https://genevestigator.com/gv/doc/intro_plant.jsp) (Hruz et al., 2008). Microarray data of UV-C treated rice were obtained from the transcriptomic analysis conducted by Park et al. (2013). The genes that changed more than two-fold, with a *p* < 0.05, were identified as being differentially expressed genes. The normalized data was uploaded and heatmap expression patterns were generated using the Multi Experiment Viewer program (http://mev.tm4.org/#/welcome).

### RNA isolation and quantitative real-time PCR analysis

Total RNA extraction from rice samples and cDNA synthesis were accomplished using the methods described above. Quantitative real-time PCR (qRT-PCR) was performed using a Prime Q-Mastermix (GeNet Bio, Daejeon, Korea) on a Rotor-Gene Q instrument system (Qiagen). For normalization of transcript levels, rice ubiquitin5 (*UBQ5*) gene (Os01g22490) was used as a reference gene, which expresses stably in rice (Jain et al., 2006). The _ΔΔ_Ct method was applied to calculate expression levels (Choi et al., 2014). To ensure primer specificity, we used the data when the melting curve showed a single peak. Primers for qRT-PCR are listed in Supplementary Table 2. The primer sequences for *OsCCR19, 20, 21*, and *UBQ5* were followed by Koshiba et al. (2013). To assess the expression of *OsCCR17, 19, 20*, and *21* in different tissues and stress conditions, qRT-PCR analysis was performed on triplicated biological samples, and each sample was analyzed twice for technical replicate. The results represent the mean ± standard deviation. One-way ANOVA and Tukey's HSD *post-hoc* test for qRT-PCR data were performed and significant differences (*p* < 0.05) were represented with the letters a and b. All statistical analysis was carried out using SPSS statistics.

## Results

### The CCR gene family in rice

*CCR*s are a large gene family in plants, and belong to the mammalian 3β-hydroxysteroid dehydrogenase (HSD)/plant dihydroflavonol reductase (DFR) superfamily (Lacombe et al., 1997; Barakat et al., 2011). In the MSU RGAP Database (Kawahara et al., 2013), 33 genes were annotated as CCRs or CCR-like (CCR/DFR/epimerase 3β-HSD) proteins in the rice genome (Table 1). The open reading frame (ORF) and peptide lengths of functional CCR genes from *A. thaliana*, maize, wheat, switchgrass and *E. gunnii* are 999–1,125 nucleotides and 332–374 amino acids long, respectively (Lacombe et al., 1997; Pichon et al., 1998; Lauvergeat et al., 2001; Ma, 2007; Escamilla-Treviño et al., 2010). Of the 33 *OsCCR*s, 24 *OsCCR*s had ORFs of a comparable size (960–1,140 nucleotides) to known functional CCR genes, which encode 319–379 amino acids (Table 1), indicating that there is no C-terminal extension in OsCCRs. These OsCCRs contained highly homologous sequences to the characteristic NAD(P)-binding and catalytic domains of CCR proteins (Supplementary Figure 1). This finding agreed well with the rice *CCR* gene family previously identified by a homology search (Kawasaki et al., 2006). Therefore, the naming of *OsCCR*s identified in this study (*OsCCR1*–*8, 10*–*24*, and *26*) followed that of Kawasaki et al. (2006). The other *OsCCR*s had short ORFs encoding <229 amino acids, and lacked one or both conserved regions (Table 1 and Supplementary Figure 1). These short *OsCCR*s were designated as *OsCCR27*-*35* (Table 1).

**Table 1:** Rice *CCR* and *CCR-like* gene family^a^.

**Locus ID**	**Name**	**Gene description**	**ORF^b^**	**Protein size (aa)^c^**	**Theoretical MW^d^ (kDa)**
Os01g18110	OsCCR4	Cinnamoyl CoA reductase, putative, expressed	981	326	36.2
Os01g18120	OsCCR5	Cinnamoyl CoA reductase, putative, expressed	987	328	36.5
Os01g45200	OsCCR2	Cinnamoyl-CoA reductase-related, putative, expressed/dihydroflavonol-4-reductase	1,092	363	39.5
Os01g61230	OsCCR6	Cinnamoyl-CoA reductase family/dihydroflavonol-4-reductase	981	326	35.8
Os01g74660	OsCCR26	Cinnamoyl-CoA reductase family/dihydroflavonol-4-reductase	984	327	35.2
Os02g08420	OsCCR21	Cinnamoyl CoA reductase, putative, expressed/dihydroflavonol-4-reductase	1,035	344	37.9
Os02g56460	OsCCR1	Cinnamoyl CoA reductase/dihydroflavonol-4-reductase	1,017	338	37.4
Os02g56680	OsCCR12	Cinnamoyl CoA reductase/dihydroflavonol-4-reductase	1,014	337	37.3
Os02g56690	OsCCR13	Cinnamoyl CoA reductase/dihydroflavonol-4-reductase	1,065	354	38.5
Os02g56700	OsCCR10	Cinnamoyl CoA reductase/dihydroflavonol-4-reductase	1,020	339	37.6
Os02g56720	OsCCR11	Cinnamoyl CoA reductase/dihydroflavonol-4-reductase	1,005	334	37
Os03g60279	OsCCR27	Cinnamoyl-CoA reductase family, putative, expressed	411	136	14.5
Os03g60380	OsCCR22	Cinnamoyl CoA reductase family/dihydroflavonol-4-reductase	1,005	334	35.4
Os05g50250	OsCCR23	Cinnamoyl-CoA reductase-related/dihydroflavonol-4-reductase	1,140	379	41.3
Os06g41800	OsCCR28	Cinnamoyl CoA reductase/dihydroflavonol-4-reductase	435	144	15.9
Os06g41810	OsCCR8	Cinnamoyl CoA reductase family/dihydroflavonol-4-reductase	966	321	35.2
Os06g41840	OsCCR7	Cinnamoyl CoA reductase family/dihydroflavonol-4-reductase	966	321	34.7
Os08g08500	OsCCR29	Cinnamoyl CoA reductase family/dihydroflavonol-4-reductase	690	229	24.9
Os08g17500	OsCCR18	Cinnamoyl CoA reductase/dihydroflavonol-4-reductase	1,029	342	34.9
Os08g34280	OsCCR20	Cinnamoyl CoA reductase/dihydroflavonol-4-reductase	1,086	361	38.7
Os09g04050	OsCCR17	Cinnamoyl CoA reductase/dihydroflavonol-4-reductase	1,044	347	37.9
Os09g08720	OsCCR24	Cinnamoyl CoA reductase/dihydroflavonol-4-reductase	975	324	35.9
Os09g09230	OsCCR30	Cinnamoyl CoA reductase, putative, expressed	447	148	16.5
Os09g09270	OsCCR31	Cinnamoyl CoA reductase/dihydroflavonol-4-reductase	321	106	11.5
Os09g25150	OsCCR19	Cinnamoyl CoA reductase/dihydroflavonol-4-reductase	1,074	357	38.6
Os09g31490	OsCCR15	Cinnamoyl CoA reductase family/dihydroflavonol-4-reductase	1,032	343	37.9
Os09g31498	OsCCR32	Cinnamoyl CoA reductase family/dihydroflavonol-4-reductase	633	210	22.7
Os09g31502	OsCCR16	Cinnamoyl CoA reductase family/dihydroflavonol-4-reductase	1,047	348	38.1
Os09g31506	OsCCR33	Cinnamoyl CoA reductase family/dihydroflavonol-4-reductase/epimerase 3β _HSD protein	663	220	24.1
Os09g31514	OsCCR14	Cinnamoyl CoA reductase family/dihydroflavonol-4-reductase/epimerase 3β _HSD protein	1,044	347	38.7
Os09g31518	OsCCR34	Cinnamoyl-CoA reductase, putative	501	166	18.3
Os09g31522	OsCCR35	Cinnamoyl CoA reductase/dihydroflavonol-4-reductase	384	127	13.7
Os10g42620	OsCCR3	Cinnamoyl-CoA reductase-related, putative, expressed/dihydroflavonol-4-reductase	960	319	35.4

a *Rice genes annotated as CCRs and CCR-like (CCR/DFR/epimerase 3β-HSD) proteins were retrieved from the MSU RGAP database*.

b ORF, Open reading frame;

c aa, Amino acid;

d *MW, Molecular weight*.

**Figure 1:**
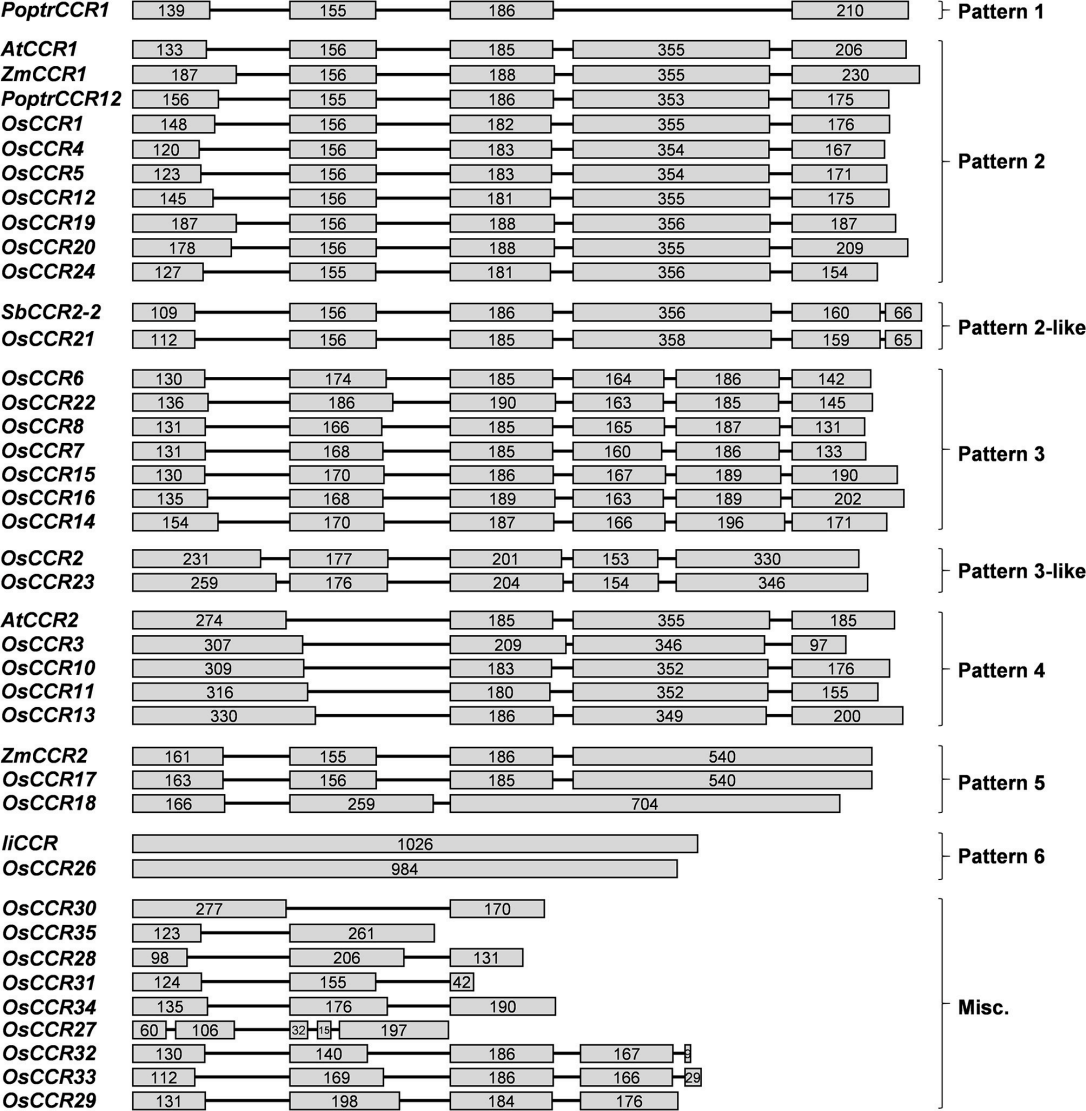
Exon-intron structures of *OsCCR*s and other plant *CCR* genes. *OsCCR*s and other plant *CCR*s were divided into nine patterns based on their exon-intron structures. Exons and introns are indicated by boxes and lines, respectively. Numbers in the boxes represent the exon sizes. The intron sizes are not to scale. The pattern names of exon-intron structures are indicated in on the right side of figure. *P. trichocarpa CCR* (*PoptrCCR*), *A. thaliana CCR* (*AtCCR*), *Z. mays CCR* (*ZmCCR*), *I. indigotica CCR* (*IiCCR*), *S. bicolor CCR* (*SbCCR*).

*OsCCR*s were distributed across rice chromosomes 1, 2, 3, 5, 6, 8, 9, and 10 (Table 1). Chromosome 9 included 12 *OsCCR*s, and chromosomes 1 and 2 contained five and six *OsCCR*s, respectively. Chromosomes 3, 5, 6, 8, and 10 contained 1–3 *OsCCR*s. These genes were composed of one to six exons (Figure 1). Based on the number of exons and exon-intron structures, Barakat et al. (2011) grouped *P. trichocarpa CCR* and *CCR-like* genes into three exon-intron patterns (Patterns 1–3). Most previously studied functional *CCR*s, such as *AtCCR1, EuCCR* (*E. gunnii CCR*), *ZmCCR1* (*Z. mays CCR1*), and *SbCCR1* (*Sorghum bicolor CCR1*), are composed of five exons, as in the exon-intron structure Pattern 2 (Lacombe et al., 1997; Lauvergeat et al., 2001; Tamasloukht et al., 2011). Of the rice genes studied in this study, *OsCCR1, 4, 5, 12, 19, 20*, and *24* had five exons, with a Pattern 2 exon-intron structure, and *OsCCR6, 7, 8, 14, 15, 16*, and *22* had six exons as in Pattern 3. Although *OsCCR21* had six exons, its exon-intron structure was more similar to that of Pattern 2; we term this Pattern 2-like (Figure 1). The exon-intron structures of *OsCCR2* and *23* were similar to Pattern 3. *OsCCR3, 10, 11*, and *13* consisted of four exons, although they had a different exon-intron pattern than Pattern 1. We designated this group as Pattern 4 (Figure 1). *OsCCR17* and *18* had exceptionally long exons, with the last exons consisting of 540 and 704 nucleotides, respectively, and were grouped into Pattern 5 (Figure 1). *OsCCR26* was composed of one exon encoding a polypeptide of 327 amino acids, which was designated Pattern 6. Although an activity assay was not performed, *IiCCR* was identified from *Isatis indigotica* and its genomic sequence was found to have no intron (Hu et al., 2011). The other *OsCCR*s were composed of two to five exons with much shorter ORF lengths than those of functional *CCR* genes (Figure 1).

### Sequence homology and phylogenetic analysis of OsCCRs

**Figure 2:**
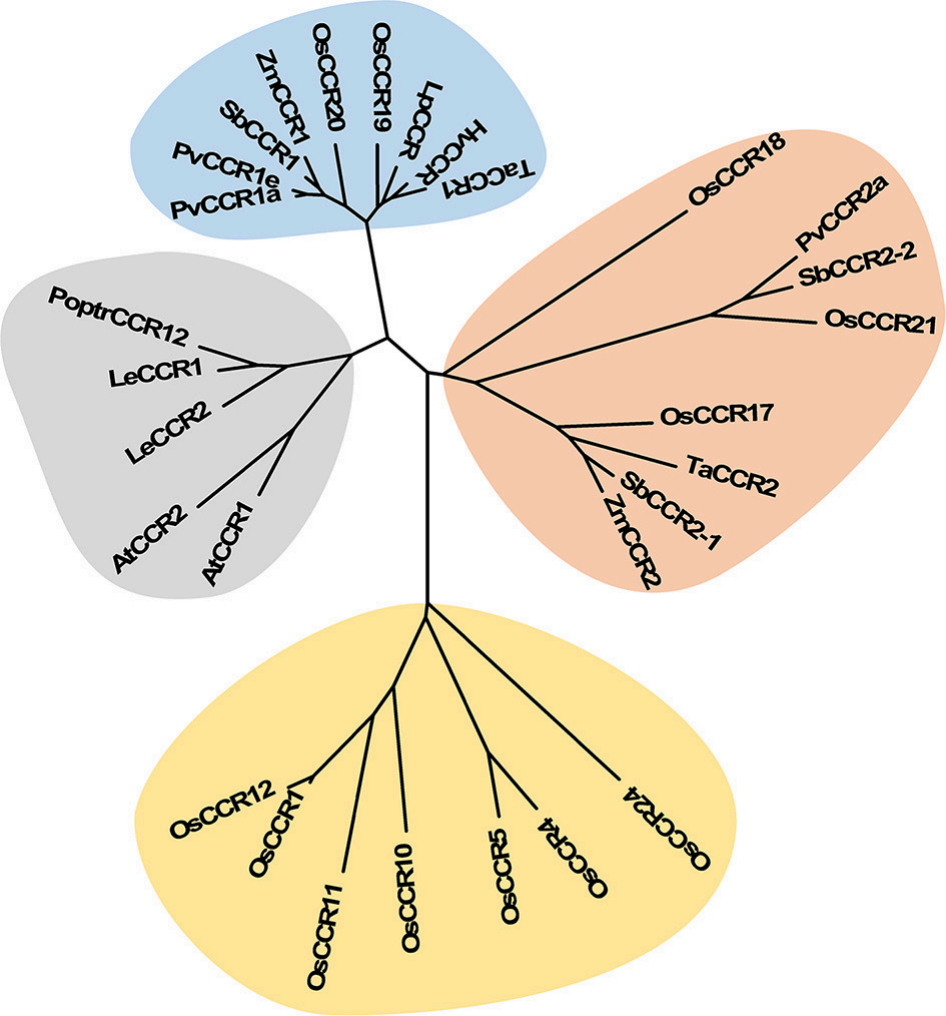
Phylogenetic analysis of OsCCRs belonging to the clade 1 of Class I (Barakat et al., 2011) and 17 characterized CCRs from other plant species. The neighbor-joining tree was built using MEGA6. The gray shade indicates dicot CCRs, and other shades indicate monocot CCRs. Blue and orange shades indicate the functional CCRs involved in developmental lignification and defense-related processes, respectively. *A. thaliana* CCRs (AtCCR1, AAG46037; AtCCR2, AAG53687); *H. vulgare* CCR (HvCCR, AAN71760); *L. esculentum* CCRs (LeCCR1, AAY41879.1; LeCCR2, AAT41880.1); *L. perenne* CCR (LpCCR, AAG09817.1); *P. trichocarpa* CCR12 (PoptrCCR12, CAA12276.1); *P. virgatum* CCRs (PvCCR1a, GQ450297; PvCCR1e, GQ450301; PvCCR2a, GQ450302); *S. bicolor* CCRs (SbCCR1, XP002445566.1; SbCCR2-1, EER98579.1; SbCCR2-2, EES04640.1); *T. aestivum* CCRs (TaCCR1, ABE01883; TaCCR2, AY771357); *Z. mays* CCRs (ZmCCR1, CAA74071; ZmCCR2, NP_001005715).

Multiple aignments of CCR protein sequences revealed that OsCCR1–25 had about 30–90% similarity to functional CCRs from other plant species (Supplementary Table 3). In particular, OsCCR20, 21, 19, and 17 were highly homologous (62–92% similarity) with known CCRs. The short length OsCCR27–35 had a low sequence homology at <39% similarity to other plant CCRs (Supplementary Table 3). A phylogenetic analysis showed that OsCCR19, 20, 17, 18, and 21 were grouped with known plant CCRs (Figure 2 and Supplementary Figure 2). In particular, OsCCR20 and 19 were closely related to PvCCR1 (*P. virgatum* CCR1), SbCCR1, ZmCCR1, LpCCR (*Lolium perenne* CCR), HvCCR (*Hordeum vulgare* CCR) and TaCCR1 (*T. aestivum* CCR1). These CCRs have been suggested as monocot functional CCRs involved in developmental lignification (Figure 2 and Supplementary Figure 2) (Pichon et al., 1998; Larsen, 2004a,b; Ma, 2007; Escamilla-Treviño et al., 2010; Tamasloukht et al., 2011; Li et al., 2016). OsCCR17, 18, and 21 were grouped with ZmCCR2, SbCCR2, TaCCR2, and PvCCR2, suggesting that they play a role in defense-related processes under biotic and abiotic stresses (Figure 2 and Supplementary Figure 2) (Pichon et al., 1998; Escamilla-Treviño et al., 2010; Li et al., 2016).

**Figure 3:**
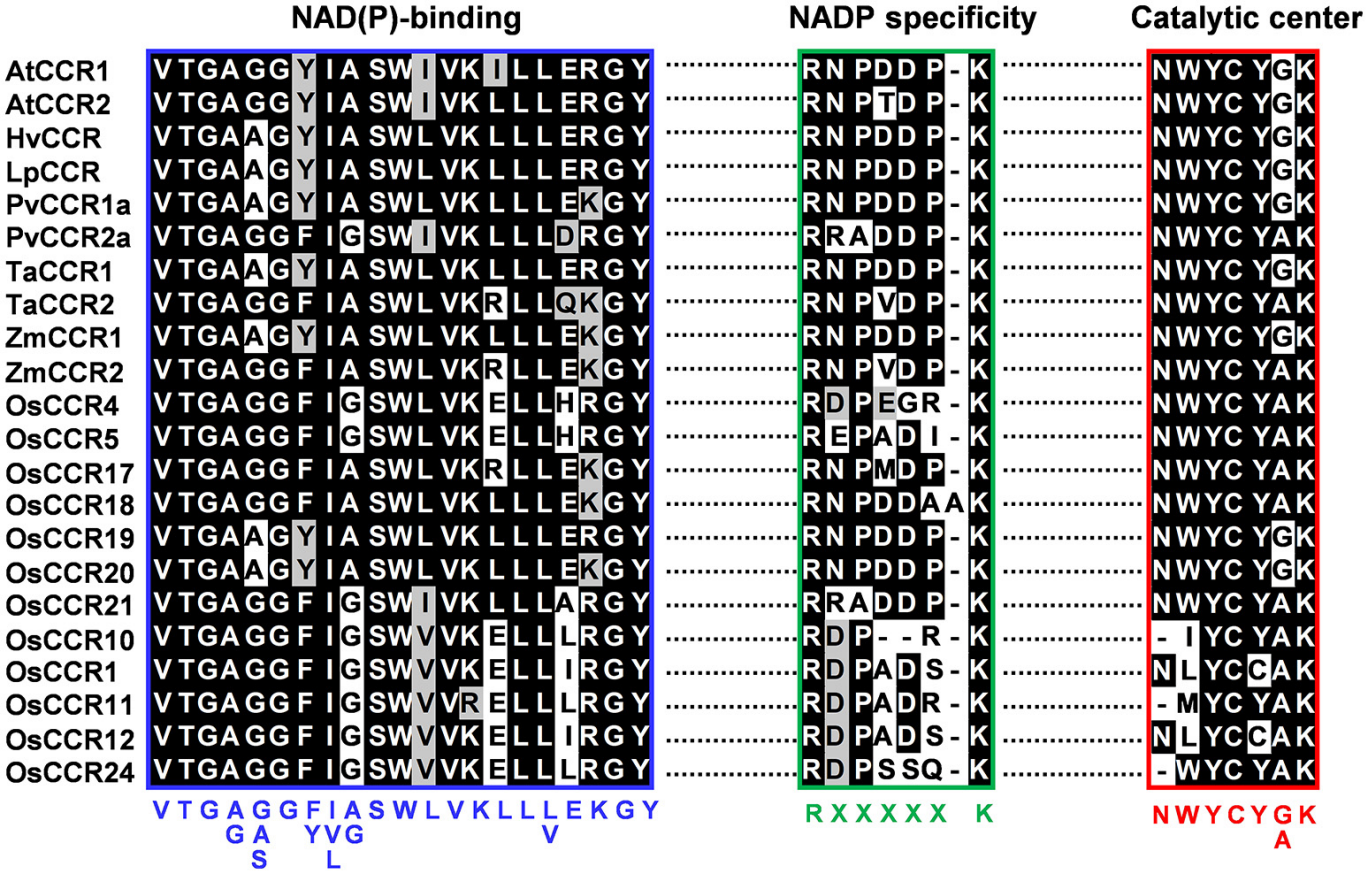
Multiple alignments of the NAD(P)-binding, NADP specificity and CCR catalytic motifs of OsCCRs belonged to the clade 1 of Class I (Barakat et al., 2011) with functional CCRs from other plant species. Amino acid sequences were aligned using Clustal-W. Shaded amino acids denote identical or similar amino acids. NAD(P)-binding and NADP specificity motifs are boxed in blue and green, respectively. The catalytic signature motif of CCRs are boxed in red. Consensus amino acid sequences are displayed below the boxes. *A. thaliana* CCRs (AtCCR1, AAG46037; AtCCR2, AAG53687); *H. vulgare* CCR (HvCCR, AAN71760); *L. perenne* CCR (LpCCR, AAG09817.1); *P. virgatum* CCRs (PvCCR1a, GQ450297; PvCCR2a, GQ450302); *T. aestivum* CCRs (TaCCR1, ABE01883; TaCCR2, AY771357); *Z. mays* CCRs (ZmCCR1, CAA74071; ZmCCR2, NP_001005715).

The most striking homology between the predicted peptide sequences of OsCCRs and functional CCRs was found in regions covered by two highly conserved motifs. These were the NAD(P)-binding motif at the N-terminus, and the catalytic motif for CCR activity (Supplementary Figure 1) (Lacombe et al., 1997; Barakat et al., 2011; Chao et al., 2017). The former is a well-conserved motif for cofactor binding in the mammalian 3β-HSD/plant DFR protein superfamily (Baker et al., 1990; Baker and Blasco, 1992; Lacombe et al., 1997; Chao et al., 2017). The latter is a CCR signature motif (NWYCYGK), in which the NWYCY sequence may be crucial for CCR activity (Lacombe et al., 1997; Escamilla-Treviño et al., 2010; Barakat et al., 2011; Chao et al., 2017). Barakat et al. (2011) applied maximum likelihood phylogenetic analysis to CCRs and CCR-like proteins from several land plant species, including *A. thaliana*, rice, *P. trichocarpa*, sorghum, grape (*Vitis vinifera*) and alfalfa (*Medicago truncatula*), and divided these proteins into three classes (Classes I–III). Class I was subdivided into three clades (clades 1–3), with the clade 1 containing functional CCRs. Most rice CCRs belonged to Class I, with OsCCR1, 4, 5, 10, 11, 12, 17, 18, 19, 20, 21, and 24 falling into the clade 1 of Class I (Barakat et al., 2011). Among the OsCCRs belonging to the clade 1 of Class I, OsCCR4, 5, 17, 18, 19, 20, and 21 contained both conserved motifs (Figure 3). The NWYCYGK motif was fully conserved in OsCCR19 and 20 (Figure 3). One amino acid variation was found in OsCCR4, 5, 21, 17, and 18, a substitution of the similar amino acid A for G in the NWYCYGK sequence. This variation also occurred in PvCCR2a, SbCCR2-1, SbCCR2-2, and ZmCCR2 (Figure 3). All other OsCCRs besides the Class I-clade 1 OsCCRs had neither one nor both conserved motifs (Supplementary Figure 1). A phylogenetic analysis also showed that OsCCR19, 20, 17, 18, and 21 were grouped with functional plant CCRs (Figure 2). OsCCR1, 4, 5, 10, 11, 12, and 24 were clearly separated from functional CCRs (Figure 2). Overall, these results suggest that OsCCR4, 5, 17, 18, 19, 20, and 21 were likely candidates for functional CCRs in rice, with OsCCR17, 18, 19, 20, and 21 being the most plausible candidates.

### Cloning and heterologous expression of *OsCCR*s

**Figure 4:**
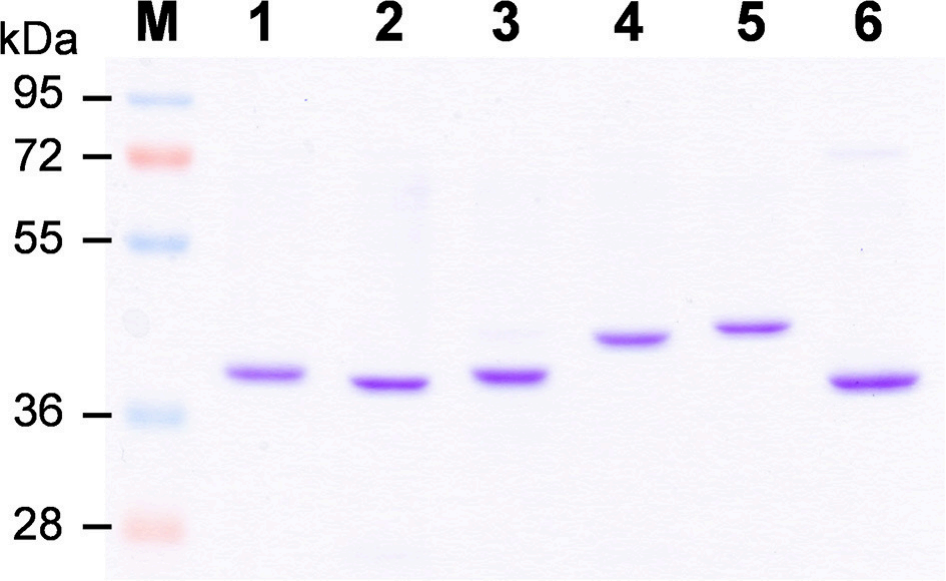
Purification of recombinant OsCCRs expressed in *E.coli*. The His-tagged OsCCR1, 5, 19, 20, 21, and 26 were expressed in *E. coli* as a soluble form. The recombinant proteins were purified by Ni^2+^-affinity chromatography. M, Molecular weight marker; 1, OsCCR1; 2, OsCCR5, 3, OsCCR19; 4, OsCCR20; 5, OsCCR21; 6, OsCCR26.

To elucidate biochemical functions of OsCCRs, we attempted to clone the likely functional candidates (*OsCCR*4*, 5, 17, 18, 19, 20*, and *21*) from wild type rice plants. The cDNAs of *OsCCR5, 17, 19, 20*, and *21* were successfully cloned from rice leaves. Despite many attempts, the cDNAs of *OsCCR4* and *18* could not be cloned from rice, which was likely a result of very low expression levels of these genes throughout all developmental stages (Supplementary Figure 3). *OsCCR1* and *26* were also cloned to examine their CCR activity. Heterologous expressions of the His-tagged OsCCR proteins were attempted under various growth temperatures and IPTG concentrations. OsCCR1, 5, 19, 20, 21, and 26 were successfully expressed as soluble protein in *E. coli* by 0.1 mM IPTG at an induction temperature of 25°C. Only limited amounts of OsCCR17 soluble proteins were expressed at 18 and 25°C, with most expressed proteins being in an insoluble form. Recombinant OsCCRs were purified with Ni^2+^ affinity chromatography to apparent homogeneity (Figure 4). The purified OsCCR proteins exhibited molecular masses of 40.5–46.4 kDa on SDS-PAGE, which agreed well with their theoretical molecular masses (Figure 4 and Table 1).

### CCR activity and kinetic parameters of the recombinant OsCCRs

To investigate the enzymatic properties of OsCCRs, the activities of recombinant OsCCRs were assayed using *p*-coumaroyl-, feruloyl-, and sinapoyl-CoAs, precursors for the H-, G-, and S-units of lignin, respectively. OsCCR17, 19, 20, and 21 showed the reductase activity to the examined substrates (Supplementary Table 4). In these OsCCRs, the NAD(P)-binding and catalytic motifs were fully conserved (Figure 3). OsCCR1, 5, and 26 showed no detectable activity toward the hydroxycinnamoyl-CoA substrate (Supplementary Table 4). In OsCCR1 and OsCCR26, the signature NWYCY motif essential for CCR activity was replaced by NLYCC and KWYPV, respectively (Figure 3). Although OsCCR5 contained a fully conserved catalytic motif, it had no detectable activity toward the hydroxycinnamoyl-CoA substrate. This was likely caused by a polymorphism in the corresponding residue of H208, which is important in substrate binding as identified by a functional analysis of PtoCCRs (*P. tomentosa* CCRs) (Supplementary Figure 1) (Chao et al., 2017). The polymorphism of H208 to A, R, M, V, K, L, M, and P residues are found in OsCCRs. This likely occurred by various duplication and retention events in CCR gene family during the evolution (Barakat et al., 2011). In OsCCR5, H208 was replaced by an R residue (Supplementary Figure 1). Of the likely functional OsCCRs, OsCCR4, and 18 had well-conserved NWYCY motif (Figure 3). OsCCR4, however, featured an H208R replacement similar to that observed in OsCCR5 (Supplementary Figure 1). OsCCR18 included one amino acid insertion (RNPDDAAK) in the NADP specificity motif [R(X)_5_K]. In functional CCRs, this motif includes five amino acids between R and K residues and forms a short loop. The R and K residues form salt bridges with the phosphate in NADPH. This motif is important in distinguishing CCR from NAD(H)-dependent short-chain dehydrogenase/reductases (SDRs) (Figure 3) (Pan et al., 2014; Chao et al., 2017). Chao et al. (2017) suggested that mutations of this motif cause the loss of enzyme activity of PtoCCR8. Therefore, we speculate that neither OsCCR4 nor 18 have any CCR activity.

To elucidate the enzymatic properties of OsCCRs, the kinetic parameters of the recombinant OsCCR19, 20, and 21 catalyzed reactions were determined toward the hydroxycinnamoyl-CoA substrate (Table 2). Although OsCCR17 displayed enzyme activity, the amount of purified proteins from the *E. coli* culture was too small for kinetic analysis. The *K*_M_-values of OsCCR20 for *p*-coumaroyl-, feruloyl-, and sinapoyl-CoA were 24.08, 15.71, and 23.34 μM, respectively (Table 2). The *k*_cat_/*K*_M_-values of OsCCR20 for feruloyl-CoA (1.41 μM^−1^ min^−1^) was about five-fold higher than those for *p*-coumaroyl- and sinapoyl-CoAs (0.32 and 0.24 μM^−1^ min^−1^, respectively), indicating that it has a greater catalytic efficiency toward feruloyl-CoA than toward the other substrates (Table 2). The *K*_M_-values of OsCCR21 for *p*-coumaroyl-, feruloyl- and sinapoyl-CoA were 16.36, 2.70, and 10.20 μM, respectively, indicating that OsCCR21 has a higher substrate affinity toward feruloyl-CoA than the other substrates (Table 2). The *k*_cat_/*K*_M_-values also revealed a greater catalytic efficiency of OsCCR21 toward feruloyl-CoA (0.77 μM^−1^ min^−1^) than toward *p*-coumaroyl- or sinapoyl-CoAs (0.08 and 0.07 μM^−1^ min^−1^, respectively). This result indicates that among three hydroxycinnamoyl-CoA substrates, both OsCCR20 and 21 have substrate preferences for feruloyl-CoA. The substrate preferences of both OsCCR20 and 21, with the strongest preference being toward feruloyl-CoA, is consistent with the lignin composition of rice. Gui et al. (2011) reported that rice lignin is composed of 70, 20, and 10% of G-, S-, and H-units, respectively. We also analyzed the lignin contents in stems of the Dongjin rice cultivar used in this study and found that the lignin composition was 62, 35, and 3% of G-, S-, and H-units, respectively (Supplementary Table 5). The *K*_M_-values of OsCCR19 for *p*-coumaroyl-, feruloyl-, and sinapoyl-CoA were 36.66, 26.85, and 62.54 μM, respectively (Table 2). OsCCR19 showed similar catalytic efficiency toward *p*-coumaroyl-, feruloyl-, and sinapoyl-CoAs with the *k*_cat_/*K*_M_-values of 0.60, 0.43, and 0.55 μM^−1^ min^−1^, respectively (Table 2).

**Table 2:** Kinetic parameters of recombinant OsCCR19, 20 and 21 catalyzed reaction with hydroxycinnamoyl-CoAs^a^.

**OsCCR**	**Substrate**	***K*_M_ (μM)**	***V*_max_ (μmol min^−1^mg^−1^)**	***k*_cat_ (min^−1^)**	***k*_cat_/*K*_M_ (μM^−1^ min^−1^)**
OsCCR19	*p*-Coumaroyl-CoA	36.66 ± 3.06	0.51 ± 0.11	21.17	0.60
	Feruloyl-CoA	26.85 ± 6.68	0.28 ± 0.12	11.48	0.43
	Sinapoyl-CoA	62.54 ± 14.08	0.83 ± 0.04	34.19	0.55
OsCCR20	*p*-Coumaroyl-CoA	24.08 ± 3.05	0.17 ± 0.02	7.72	0.32
	Feruloyl-CoA	15.71 ± 1.55	0.58 ± 0.12	22.61	1.41
	Sinapoyl-CoA	23.34 ± 2.60	0.13 ± 0.01	5.64	0.24
OsCCR21	*p*-Coumaroyl-CoA	16.36 ± 4.69	0.02 ± 0.003	1.12	0.08
	Feruloyl-CoA	2.70 ± 1.69	0.03 ± 0.003	1.59	0.77
	Sinapoyl-CoA	10.20 ± 1.84	0.02 ± 0.004	0.72	0.07

a *The results represent the mean ± standard deviation of four independent experiments*.

### *In silico* and qRT-PCR analyses of *OsCCR* gene expression

**Figure 5:**
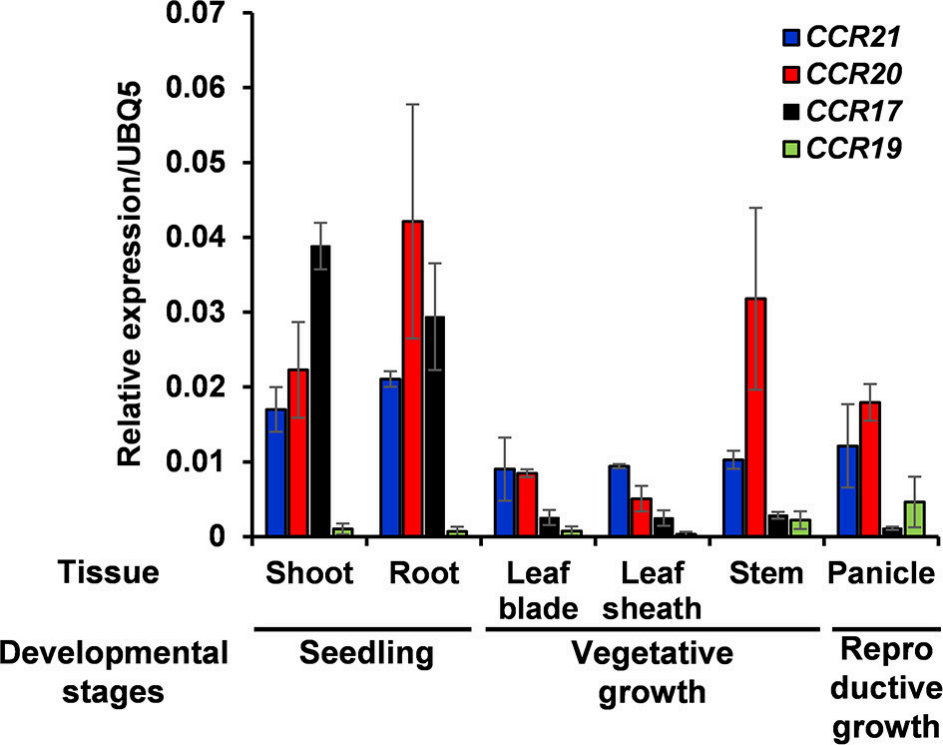
Quantitative real-time PCR analysis of *OsCCR17, 19, 20*, and *OsCCR21* gene expression in rice seedlings and different organs. Root and shoot samples were collected from 10-day old rice seedlings. Ten-week old rice plants yielded leaf, leaf sheath and stem samples. Panicles were obtained from 14-week old rice plants. An ubiquitin gene (*OsUBQ5*) was amplified using specific primers and used as an internal control. Expression levels of each *OsCCR* gene are presented as the relative expression compared to the *OsUBQ5* mRNA level. qRT-PCR analysis was performed on the triplicated biological samples. The results represent the mean ± standard deviation.

Expression of *OsCCR*s were investigated with the microarray data obtained from the Genevestigator database. Some *OsCCR*s (*OsCCR3, 6, 7, 8, 16, 19, 20, 21, 22*, and *26*) displayed a high level of expression throughout all developmental stages including germination, seedling, tillering, stem elongation, booting, heading, flowering, milk, and dough stages (Supplementary Figure 3). Of these constitutively expressed *OsCCR*s, *OsCCR19, 20*, and *21* were found to encode biochemically active CCRs (Table 2 and Supplementary Table 4), suggesting that these genes likely play a physiological role in rice. The *OsCCR17* gene encoding enzymatically active CCR was expressed only during the early growth stages (Supplementary Figure 3). Expression of *OsCCR17, 19, 20*, and *21* in different developmental stages and tissues of rice were also examined by qRT-PCR analysis. Similar with the microarray data, *OsCCR20* and *21* were expressed in all examined stages and tissues, and *OsCCR17* was expressed primarily in rice seedling shoots and roots (Figure 5). Although both *OsCCR20* and *21* were constitutively expressed in the examined rice tissues, the *OsCCR20* expressions were much higher in actively lignifying organs, such as roots and stems than those of *OsCCR21*. qRT-PCR analysis showed that expression of *OsCCR19* was very low in most examined rice tissues (Figure 5). The phylogenetic analysis revealed that OsCCR19 and 20 was closely related to functional CCRs, including ZmCCR1, PvCCR1, LpCCR, and HvCCR. These CCRs may participate in developmental lignin deposition in secondary cell walls (Larsen, 2004a,b; Escamilla-Treviño et al., 2010; Tamasloukht et al., 2011). This evidence suggests that *OsCCR*20 acts as a functional rice CCR and involved in developmental lignification.

**Figure 6:**
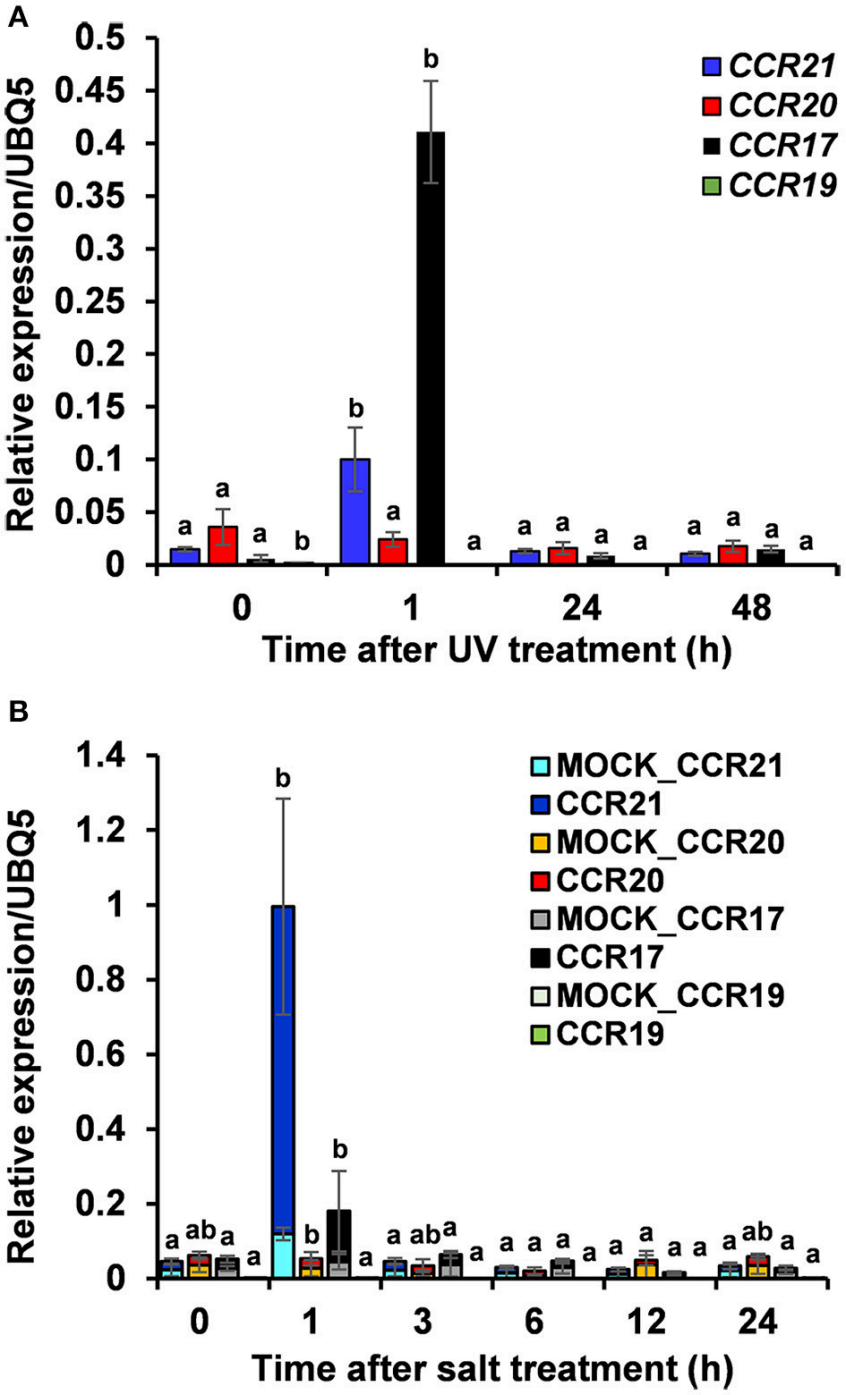
Quantitative real-time PCR analysis of *OsCCR17, 19, 20* and *OsCCR21* gene expression in response to UV-irradiation and salt treatment. Rice plants treated with UV **(A)** and salt **(B)** were collected at designated time points and used to examine the expression of *OsCCR17, 19, 20*, and *OsCCR21*. An ubiquitin gene (*OsUBQ5*) was amplified using specific primers and used as an internal control. Expression level of each *OsCCR* gene was presented as the relative expression compared to the *OsUBQ5* mRNA level. qRT-PCR analysis was performed on the triplicated biological samples. One-way ANOVA and Tukey's HSD *post-hoc* test for qRT-PCR data were performed and significant differences (*p* < 0.05) were represented with the letters a and b. The results represent the mean ± standard deviation.

Expression profiles of *OsCCR*s were also altered by biotic and abiotic stresses. Transcriptomic analysis showed that expression of *OsCCR*s was significantly induced during abiotic stress conditions, such as exposure to cold (*OsCCR1, 2, 6, 21, 23*, and *33*), drought (*OsCCR3, 7*, and *15*), and high salinity (*OsCCR3, 7, 17*, and *18*) (Supplementary Figure 4A). Under abiotic stress conditions, the biochemically functional genes *OsCCR21* and *17* were induced by cold and salt stresses, respectively. In our previous microarray data of UV-treated rice leaves (Park et al., 2013), *OsCCR1, 3, 17, 20, 21*, and *23* were found to be up-regulated in response to UV-irradiation (Supplementary Figure 4B). *In silico* analysis of public microarray data also showed that the expression of several *OsCCR*s was up-regulated by biotic stresses, such as *M. grisea, Xoo*, and *Xoc* infections. The expression of *OsCCR1, 2, 3, 5, 17, 18, 20*, and *21* were induced by *M. grisea* infection. The expression of functional *OsCCR17, 20*, and *21* were induced by both UV-irradiation and *M. grisea* infection. Infection with *Xoo* stimulated the expression of *OsCCR20* and *21*, and the *Xoc* infection induced *OsCCR21* expression (Supplementary Figure 5). Among these stress-inducible *OsCCR*s, expression of *OsCCR17* and *21* was frequently observed to be stimulated by multiple abiotic stresses. To confirm the stress-inducible expression of *OsCCR*s, qRT-PCR analysis was performed with UV-treated rice leaves and salt-treated rice seedlings (Figure 6). The expression level of *OsCCR17* and *21* in UV-treated rice leaves increased about 70- and 10-fold compared to those of the non-treated control an hour after UV treatment, respectively (Figure 6A). The expressions of *OsCCR17* and *21* were also significantly increased by salt treatment compared to a control, which received a mock treatment (Figure 6B). The qRT-PCR analysis showed that the transcript levels of *OsCCR19* and *20* were not significantly changed by both stress conditions (Figure 6). These results suggest that *OsCCR17* and *21* are most likely involved in the stress responses of rice.

## Discussion

Although plant CCRs comprise a large gene family, only a small number of CCR genes have been reported to encode biochemically active CCRs for the biosynthesis of lignin and defense-related phenolic compounds (Lauvergeat et al., 2001; Costa et al., 2003; Escamilla-Treviño et al., 2010; Barakat et al., 2011). Xu et al. (2009) suggested that the expansion of lignin biosynthetic gene families was rapidly occurred after divergence of monocots and dicots at 120 million years ago. The large gene family of *CCR*s in plants was suggested to occur by various duplication and retention events during the evolution and indeed, 67% of rice *CCR* and *CCR-like* genes were located on duplicated chromosome regions (Barakat et al., 2011). A large member of CCR gene family in plants has also been supposed to because of their substrate diversity (Xu et al., 2009). All previously characterized CCRs showed similar peptide lengths ranging from 332 to 374 amino acids in *A. thaliana*, wheat, sorghum, switchgrass, and *E. gunnii* (Lacombe et al., 1997; Lauvergeat et al., 2001; Ma, 2007; Escamilla-Treviño et al., 2010; Li et al., 2016). Twenty-four OsCCRs had peptide lengths similar to known CCRs (Table 1). These OsCCRs showed high homology to well-conserved NAD(P)-binding and catalytic motifs of functional CCRs (Supplementary Figure 1 and Table 1). As a member of the mammalian 3β-HSD/plant DFR superfamily, CCRs share the NAD(P)-binding domain with DFRs. CCRs, however, have the distinct catalytic motifs with signature NWYCYGK sequence different from DFRs (Lacombe et al., 1997; Escamilla-Treviño et al., 2010; Barakat et al., 2011; Chao et al., 2017). Of OsCCRs with appropriate peptide lengths, OsCCR19 and 20 exhibited the fully conserved catalytic motif, and OsCCR4, 5, 17, 18, and 21 had the signature motif with one amino acid variation (G to A) (Figure 3). The G to A variation in the CCR catalytic motifs has been frequently found in other active CCRs, such as PvCCR2a and ZmCCR2 (Figure 3) (Pichon et al., 1998; Escamilla-Treviño et al., 2010; Li et al., 2016). Indeed, our biochemical assays confirmed that OsCCR17, 19, 20 and 21 had CCR activity to hydroxycinnamoyl-CoAs (Table 2 and Supplementary Table 4). OsCCR1 and 26, with two and four mismatches in this motif, respectively, had no CCR activity (Supplementary Table 4). This evidence suggests that the NWYCY(G/A)K sequence is crucial for CCR activity. An activity assay also revealed that OsCCR5 had no CCR activity, although it contained the signature catalytic motif. A recent study demonstrated that H208 in PtoCCRs is indispensable for substrate binding, and is conserved in the functional CCRs from other plant species (Supplementary Figure 1) (Chao et al., 2017). In OsCCR5, H208 was replaced by R, which likely caused the loss of its CCR activity. Like OsCCR5, OsCCR4 had the H208R replacement. In addition to the NAD(P)-binding motif, the NADP-specific R(X)_5_K motif was identified by structural analysis of *M. truncatula* CCR2 and petunia CCR1. This NADP-specificity motif is a key structure distinguishing CCRs from NAD(H)-dependent SDRs (Pan et al., 2014). This motif was well-conserved in the active OsCCRs (Figure 3). OsCCR18 showed one amino acid insertion in the NADP specificity motif (Figure 3). Although no activity assay was performed, for these reasons, we speculate that OsCCR4 and 18 had no CCR activity. Altogether, this evidence suggested that of the 33 OsCCRs studied here, OsCCR17, 19, 20, and 21 may encode biochemically functional CCRs in rice. In addition, a previous study reported that the enzyme activity of OsCCR1 is activated by the small GTPase *OsRac1* that controls defense-related lignin synthesis (Kawasaki et al., 2006).

Plant *CCR* and *CCR-like* genes are composed of different numbers of exons and exon-intron structures. The *A. thaliana CCR* gene family has been suggested to have seven patterns of exon-intron structures (Barakat et al., 2011). The *OsCCR*s examined in this study also exhibited eight exon-intron patterns (Figure 1). Barakat et al. (2011) divided the exon-intron structures of *PoptrCCR*s into three patterns (Patterns 1–3) comprised of 4, 5, and 6 exons, respectively. In Pattern 2, the fourth exon is about two times longer than other exons. The length of the fourth exon in Pattern 2 is similar to the combined lengths of the fourth and fifth exons of Pattern 3 (Barakat et al., 2011). Most functional *CCR*s, such as *AtCCR1, EuCCR, ZmCCR1*, and *SbCCR1*, involved in developmental lignification are grouped into Pattern 2 (Figure 1) (Lacombe et al., 1997; Lauvergeat et al., 2001; Tamasloukht et al., 2011). Consistently, *OsCCR19* and *20* were composed of five exons with the exon-intron structure of Pattern 2. Although *OsCCR21*, which encoded biochemically active CCR, had six exons, the exon-intron structure differed from that of Pattern 3. Rather, the exon-intron structure of *OsCCR21* was more similar to Pattern 2 (Pattern 2-like), with the length of the fourth exon equaling that seen in Pattern 2 (Figure 1). *SbCCR2-2* has been reported to also exhibit a Pattern 2-like exon-intron structure (Figure 1) (Li et al., 2016). The biochemically active *OsCCR17* was composed of four exons with an exceptionally long fourth exon (Pattern 5) (Figure 1). *ZmCCR2* showed the exon-intron structure of Pattern 5 (Pichon et al., 1998). *AtCCR2* exhibited the exon-intron structure of Pattern 4, being composed of four exons (Figure 1) (Lauvergeat et al., 2001). Unlike *CCR* genes involved in developmental lignification, which were mostly grouped into Pattern 2, stress-related *CCR*s such as *AtCCR2, ZmCCR2*, and *SbCCR2-2* exhibited diverse exon-intron patterns.

Enzymatic properties of CCRs have been elucidated from many plants, and observed to reflect the lignin compositions of the source plant species (Piquemal et al., 1998; Ma and Tian, 2005; Tamasloukht et al., 2011). Lignins of gymnosperm wood are predominantly composed of G-units (Campbell and Sederoff, 1996; Donaldson, 2001; Vanholme et al., 2010). Unlike gymnosperm lignins, most angiosperm lignins are a mixture of G- and S-units (Donaldson, 2001; Vanholme et al., 2010). The proportion of H-units is variable within plant species and even between tissues in the same plant (Campbell and Sederoff, 1996; Vanholme et al., 2010). Wheat CCRs (TaCCR1 and 2) and switchgrass PvCCR1 have substrate preference for feruloyl-CoA, a precursor for the G-unit (Ma and Tian, 2005; Ma, 2007; Escamilla-Treviño et al., 2010). Similarly, OsCCR20 and 21 showed a preference for feruloyl-CoA over other CoA esters (Table 2). This result agrees well with rice lignin compositions, which has a high G-unit content and a relatively small portion of S- and H-units (Gui et al., 2011 and Supplementary Table 5). Different from OsCCR20, OsCCR19 showed similar catalytic efficiency toward three examined substrates. It has been known that the substrate preferences of CCRs vary between CCRs from different plant species, even in isozymes from the same species (Goffner et al., 1994; Baltas et al., 2005; Li et al., 2005; Escamilla-Treviño et al., 2010; Tamasloukht et al., 2011).

During development, lignin is deposited in the thickened secondary cell walls. In addition, its synthesis can be induced by diverse biotic and abiotic stresses (Moura et al., 2010; Miedes et al., 2014). In *A. thaliana*, maize and switchgrass, *CCR1* genes are related to lignin biosynthesis during development and *CCR2* genes are involved in stress-related processes (Pichon et al., 1998; Lauvergeat et al., 2001; Escamilla-Treviño et al., 2010; Tamasloukht et al., 2011). Phylogenetic analysis of functional CCRs has revealed that constitutive CCRs involved in developmental lignification are grouped separately from CCRs implicated in defense-related processes (Figure 2 and Supplementary Figure 2) (Escamilla-Treviño et al., 2010; Li et al., 2016; Chao et al., 2017). OsCCR19 and 20 were closely related to constitutive CCRs, such as ZmCCR1, PvCCR1, and LpCCR (Figure 2). CCRs in this group have been observed to be highly expressed in actively lignifying tissues, including stems and roots (Pichon et al., 1998; Larsen, 2004a,b; Escamilla-Treviño et al., 2010). *In silico* transcriptomic analysis showed that *OsCCR19* and *20* are constitutively expressed throughout all developmental stages of rice (Supplementary Figure 3). Our qRT-PCR analysis also revealed strong expressions of *OsCCR20* in lignifying tissues such as roots and stems (Figure 5). Unlike the microarray data, *OsCCR19* was rarely expressed in most examined tissues. The kinetic analysis also showed that *OsCCR20* was more enzymatically efficient toward feruloyl-CoA, a precursor of the lignin G-unit (Table 2). These results suggest that *OsCCR20* primarily participates in developmental deposition of lignins in secondary cell wall. Lignification occurs prominently in differentiating xylem tissues and interfascicular fibers in stems and roots (Lacombe et al., 1997; Goujon et al., 2003; Tamasloukht et al., 2011). Functional CCRs involved in developmental lignification have been found to localize in these tissues. *In situ* hybridization of the CCR antisense probe shown that the CCR transcripts are localized in the differentiating xylem tissues of poplar stems (Lacombe et al., 1997). In *Leucaena leucocephala* seedlings, the CCR proteins are localized in the developing xylem tissues of stems and roots (Srivastava et al., 2015). Transient expression of SbCCR-GFP in tobacco leaves indicated that CCR proteins are localized in the cytoplasm (Li et al., 2016). Kawasaki et al. (2006) also reported that OsCCR1 is localized in the cytoplasm. Analysis of N-terminal sequence of OsCCRs using the SignalP tool (http://www.cbs.dtu.dk/services/SignalP/) showed that all OsCCRs, except OsCCR27, have no signal sequence. The expression of *CCR*s in other groups are induced under various stress conditions (Lauvergeat et al., 2001; Fan et al., 2006; Escamilla-Treviño et al., 2010; Li et al., 2016). For instance, *ZmCCR2* expression was highly induced by water deficit in the root elongation zone of maize (Fan et al., 2006). *PvCCR2* was highly induced after the rust disease infection (Escamilla-Treviño et al., 2010), and *SbCCR2-2* expression was stimulated by sorghum aphid infection (Li et al., 2016). Phylogenetic analysis indicated that OCCR17 and 21 were grouped with the stress-inducible CCRs (Figure 2). Although transcriptomic analysis revealed constitutive expression of *OsCCR21*, its expression was strongly stimulated by infections of rice pathogens (*M. grisea, Xoo* and *Xoc*) (Supplementary Figure 5). Expression of *OsCCR17* was also induced by *M. grisea* and *Xoo* infections. In addition, *OsCCR17* and *21* expressions were strongly induced by abiotic stresses, such as cold, high salinity, and UV-irradiation (Supplementary Figure 4). Transcriptomic analysis of UV-treated rice has revealed that a set of phenylpropanoid and monolignol pathway genes are co-expressed immediately after UV-treatment, with the response involving biosynthesis of defense-related compounds such as phytoalexins (Park et al., 2013, 2014; Cho and Lee, 2015). Our qRT-PCR analysis also observed strong induction of *OsCCR17* and *21* in response to UV and salt-treatment (Figure 5). These results strongly suggest that *OsCCR17* and *21* likely participates in defense-related lignification and synthesis of phenolic compounds.

## Conclusion

Expression patterns and biochemical properties of the rice *CCR* gene family were thoroughly analyzed in the present study. OsCCR17, 19, 20, and 21 were found to have NAD(P)-binding and NADP-specific motifs as well as the CCR signature motif. The recombinant OsCCR17, 19, 20, and 21 showed enzyme activity toward hydroxycinnamoyl-CoA substrates, indicating that these OsCCRs are biochemically functional CCRs in rice. Phylogenetic analysis revealed that OsCCR19 and 20 were closely related to other plant CCRs involved in developmental lignification. *In silico* transcriptomic analysis and qRT-PCR consistently demonstrated that *OsCCR20* were constitutively expressed throughout all developmental stages of rice, with especially high expression levels in actively lignifying tissues such as roots, stems and panicles. These results suggest that *OsCCR20* are primarily involved in the developmental deposition of lignins in secondary cell walls. Meanwhile, the expressions of *OsCCR17* and *21* were induced in response to biotic and abiotic stresses, such as *M. grisea* and *Xoo* infections, UV-irradiation and high salinity. OsCCR17 and 21 were also grouped with stress-responsible CCRs identified from other plant species. Therefore, we suggest that *OsCCR17* and *21* play a role in defense-related processes of rice under biotic and abiotic stress conditions.

## Author contributions

M-HC and S-WL: conceived and designed the experiments; HLP and M-HC: performed the experiments and conducted bioinformatics analyses; MK: performed the experiments. M-HC, HLP, SHB, and S-WL: analyzed the data, and wrote the manuscript.

### Conflict of interest statement

The authors declare that the research was conducted in the absence of any commercial or financial relationships that could be construed as a potential conflict of interest.

## References

[B1] Ahuja I. Kissen R. Bones A. M. (2012). Phytoalexins in defense against pathogens. Trends Plant Sci. 17, 73–90. 10.1016/j.tplants.2011.11.002 22209038

[B2] Baker M. E. Blasco R. (1992). Expansion of the mammalian 3β-hydroxysteroid dehydrogenase/plant dihydroflavonol reductase superfamily to include a bacterial cholesterol dehydrogenase, a bacterial UDP-galactose 4-epimerase, and open reading frames in vaccinia virus and fish lymphocystis disease virus. FEBS Lett. 301, 89–93. 10.1016/0014-5793(92)80216-4 1451793

[B3] Baker M. E. Luu-The V. Simard J. Labrie F. (1990). A common ancestor for mammalian 3β-hydroxysteroid dehydrogenase and plant dihydroflavonol reductase. Biochem. J. 269, 558–559. 10.1042/bj2690558 2201288 PMC1131618

[B4] Baltas M. Lapeyre C. Bedos-Belval F. Maturano M. Saint-Aguet P. Roissel L.. (2005). Kinetic and inhibition studies of cinnamoyl-CoA reductase 1 from *Arabidopsis thaliana*. Plant Physiol. Biochem. 43, 746–753. 10.1016/j.plaphy.2005.06.003 16122934

[B5] Barakat A. Yassin N. B. Park J. S. Choi A. Herr J. Carlson J. E. (2011). Comparative and phylogenomic analyses of cinnamoyl-CoA reductase and cinnamoyl-CoA-reductase-like gene family in land plants. Plant sci. 181, 249–257. 10.1016/j.plantsci.2011.05.012 21763535

[B6] Barber M. S. McConnell V. S. DeCaux B. S. (2000). Antimicrobial intermediates of the general phenylpropanoid and lignin specific pathways. Phytochemistry 54, 53–56. 10.1016/S0031-9422(00)00038-8 10846747

[B7] Bart R. S. Chern M. Vega-Sánchez M. E. Canlas P. Ronald P. C. (2010). Rice *Snl6*, a cinnamoyl-CoA reduactase-like gene family member, is required for NH1-mediated immunity to *Xanthomonas oryaze* pv. *oryzae*. PLoS Genet. 6:e1001123. 10.1371/journal.pgen.1001123 20862311 PMC2940737

[B8] Beuerle T. Pichersky E. (2002). Enzymatic synthesis and purification of aromatic coenzyme A esters. Anal. Biochem. 302, 305–312. 10.1006/abio.2001.5574 11878812

[B9] Bonawitz N. D. Chapple C. (2010). The genetics of lignin biosynthesis: connecting genotype to phenotype. Annu. Rev. Genet. 44, 337–363. 10.1146/annurev-genet-102209-163508 20809799

[B10] Boyer J. S. (1982). Plant productivity and environment. Science 218, 443–448. 10.1126/science.218.4571.443 17808529

[B11] Campbell M. M. Sederoff R. R. (1996). Variation in lignin content and composition (mechanisms of control and implications for the genetic improvement of plants). Plant Physiol. 110, 3–13. 10.1104/pp.110.1.3 12226169 PMC157688

[B12] Carocha V. Soler M. Hefer C. Cassan-Wang H. Fevereiro P. Myburg A. A.. (2015). Genome-wide analysis of the lignin toolbox of *Eucalyptus grandis*. New phytol. 206, 1297–1313. 10.1111/nph.13313 25684249

[B13] Chakraborty S. Newton A. C. (2011). Climate change, plant diseases and food security: an overview. Plant Path. 60, 2–14. 10.1111/j.1365-3059.2010.02411.x

[B14] Chao N. Li N. Qi Q. Li S. Lv T. Jiang X. N.. (2017). Characterization of the cinnamoyl-CoA reductase (CCR) gene faily in *Populus tomentoosa* reveals the enzymatic active sites and evolution of CCR. Planta 245, 61–75. 10.1007/s00425-016-2591-6 27580618

[B15] Cho M. H. Lee S. W. (2015). Phenolic phytoalexins in rice: biological functions and biosynthesis. Int. J. Mol. Sci. 16, 29120–29133. 10.3390/ijms161226152 26690131 PMC4691099

[B16] Choi C. C. Lee S. Kim S. R. Lee Y. S. Liu C. Cao X. (2014). Trithorax group protein *Oryzae sativa* trithorzs1 controls flowering time in rice via interaction with early heading date3. Plant Physiol. 164, 1326–1337. 10.1104/pp.113.228049 24420930 PMC3938623

[B17] Costa M. A. Collins R. E. Anterola A. M. Chchrane F. C. Davin L. B. Lewis N. G. (2003). An *in silico* assessment of gene function and organization of the phenylpropanoid pathway metabolic networks in *Arabidopsis thaliana* and limitations thereof. Phytochemistry 64, 1097–1112. 10.1016/S0031-9422(03)00517-X 14568076

[B18] Davin L. B. Jourdes M. Patten A. M. Kim K. W. Vassão D. G. Lewis N. G. (2008). Dissection of lignin macromolecular configuration and assembly: comparison to related biochemical processes in allyl/propenyl phenol and lignin biosynthesis. Nat. Prod. Rep. 25, 1015–1090. 10.1039/b510386j 19030603

[B19] Dixon R. A. Achnine L. Kota P. Liu C. J. Reddy M. S. Wang L. (2002). The phenylpropanoid pathway and plant defence – a genomics perspective. Mol. Plant Pathol. 3, 371–390. 10.1046/j.1364-3703.2002.00131.x 20569344

[B20] Donaldson L. A. (2001). Lignification and lignin topochemistry - an ultrastructural view. Phytochemistry 57, 859–873. 10.1016/S0031-9422(01)00049-8 11423137

[B21] Escamilla-Treviño L. L. Shen H. Uppalapati S. R. Ray T. Tang Y. Hernandez T. (2010). Switchgrass (*Panicum virgarum*) possesses a divergent family of cinnamoyl CoA reductase distinct biochemical properties. New Phytol. 185, 143–155. 10.1111/j.1469-8137.2009.03018.x 19761442

[B22] Fan L. Linker R. Gepstein S. Tanimoto E. Yamamoto R. Neumann P. M. (2006). Progressive inhibition by water deficit of cell wall extensibility and growth along the elongation zone of maize roots is related to increased lignin metabolism and progressive stelar accumulation of wall phenolics. Plant Physiol. 140, 603–612. 10.1104/pp.105.073130 16384904 PMC1361327

[B23] Goffner D. Campbell M. M. Campargue C. Clastre M. Borderies G. Boudet A.. (1994). Purification and characterization of cinnamoyl-Coenzyme A:NADP oxidoreductase in *Eucalytus gunnii*. Plant Physiol. 106, 625–632. 10.1104/pp.106.2.625 12232355 PMC159569

[B24] Goujon T. Ferret V. Mila I. Pollet B. Ruel K. Burlat V.. (2003). Down-regulation of the AtCCR1 gene in *Arabidopsis thaliana*: effects on phenotype, lignins and cell wall degradability. Planta 217, 218–228. 10.1007/s00425-003-0987-6 12783329

[B25] Gross G. G. (1981). The biochemistry of lignification. Adv. Bot. Res. 8, 25–63. 10.1016/S0065-2296(08)60032-4

[B26] Großkinsky D. K. van der Graaff E. Roitsch T. (2012). Phytoalexin transgenics in crop protection-Fairy tale with a happy end? Plant sci. 195, 54–70. 10.1016/j.plantsci.2012.06.008 22920999

[B27] Gui J. Shen J. Li L. (2011). Functional characterization of evolutionarily divergent 4-coumarate:coenzyme a ligases in rice. Plant Physiol. 157, 574–586. 10.1104/pp.111.178301 21807887 PMC3192572

[B28] Hamann T. (2012). Plant cell wall integrity maintenance as an essential component of biotic stress response mechanisms. Front. Plant Sci. 3:77. 10.3389/fpls.2012.00077 22629279 PMC3355559

[B29] Hruz T. Laule O. Szabo G. Wessendorp F. Bleuler S. Oertle L.. (2008). Genevestigator V3: a reference expression database for the meta-analysis of transcriptomes. Adv. Bioinform. 2008:420747. 10.1155/2008/420747 19956698 PMC2777001

[B30] Hu Y. Di P. Chen Y. Xiao Y. Zhang L. Chen W. (2011). Isolation and characterization of a gene encoding cinnamoyl-CoA reductrase from *Isatis indigotica* fort. Mol. Biol. Rep. 38, 2075–2083. 10.1007/s11033-010-0333-6 20859691

[B31] Ishihara A. Hashimoto Y. Tanaka C. Dubouzet J. G. Nakao T. Matsuda F.. (2008). The tryptophan pathway is involved in the defense responses of rice against pathogenic infection via serotonin production. Plant J. 54, 481–495. 10.1111/j.1365-313X.2008.03441.x 18266919

[B32] Ishihara A. Nakao T. Mahimo Y. Murai M. Ichimaru N. Tanaka C.. (2011). Probing the role of tryptophan-derived secondary metabolism in defense responses against *Bipolaris oryzae* infection in rice leaves by a suicide substrate of tryptophan decarboxylase. Phytochemistry 72, 7–13. 10.1016/j.phytochem.2010.11.001 21112065

[B33] Jain M. Nijhawan A. Tyagi A. K. Khurana J. P. (2006). Validation of housekeeping genes as internal control for studying gene expression in rice by quantitative real-time PCR. Biochem. Biophys. Res. Commun. 345, 646–651. 10.1016/j.bbrc.2006.04.140 16690022

[B34] Kawahara Y. de la Bastide M. Hamilton J. P. Kanamori H. McCombie W. R. Ouyang S.. (2013). Improvement of the *Oryza sativa Nipponbare* reference genome using next generation sequence and optical map data. Rice 6:4. 10.1186/1939-8433-6-4 24280374 PMC5395016

[B35] Kawasaki T. Koita H. Nakatsudo T. Hasegawa K. Wakabayashi K. Takahashi H.. (2006). Cinnamoyl-CoA reductase, a key enzyme in lignin biosynthesis, is an effector of small GTPase Rac in defense signaling in rice. Proc. Natl. Acad. Sci. U.S.A. 103, 230–235. 10.1073/pnas.0509875103 16380417 PMC1325009

[B36] Keen N. T. Littlefield L. J. (1979). The possible association of phytoalexins with resistance gene expression in flax to *Melampsora lini*. Physiol. Plant Pathol. 14, 265–280. 10.1016/0048-4059(79)90048-1

[B37] König S. Feussner K. Kaever A. Landesfeind M. Thurow C. Karlovsky P.. (2014). Soluble phenylpropanoids are involved in the defense response of *Arabidopsis* against *Verticillium longisporum*. New Phytol. 202, 823–837. 10.1111/nph.12709 24483326

[B38] Koshiba T. Hirose N. Mukai M. Yamamura M. Hattori T. Suzuki S. (2013). Characterization of 5-hydroxyconiferaldehyde *O*-methyltransferase in *Oryzae sativa*. Plant Biotechnol. 30, 157–167. 10.5511/plantbiotechnology.13.0219a

[B39] Lacombe E. Hawkins S. Doorsselaere J. V. Piquemal J. Goffner D. Poeydomenge O.. (1997). Cinnamoyl CoA reductase, the first commited enzyme of the linin branch biosynthetic pathway: colning, expression and phylogenetic relationships. Plant J. 11, 429–441. 10.1046/j.1365-313X.1997.11030429.x 9107033

[B40] Larsen K. (2004a). Cloning and characterization of a ryegrass (*Lolium perenne*) gene emcoding cinnamoyl-CoA reductase (CCR). Plant sci. 166, 569–581. 10.1016/j.plantsci.2003.09.026

[B41] Larsen K. (2004b). Molecular cloning and characterization of cDNAs encoding cinnamoyl CoA reductase (CCR) from barley (*Hordeum vulgare*) and potato (*Solanum tuberosum*). J. Plant Physiol. 161, 105–112. 10.1078/0176-1617-01074 15002670

[B42] Lauvergeat V. Lacomme C. Lacombe E. Lasserre E. Poby D. Grima-Pettenati J. (2001). Two-cinnamoyl-CoA reductase (CCR) genes from *Arabidopsis thaliana* are differentially expressed during development and in response to infection with pathogenic bacteria. Phytochemistry 57, 1187–1195. 10.1016/S0031-9422(01)00053-X 11430991

[B43] Li J. Fan F. Wang L. Zhan Q. Wu P. Du J.. (2016). Cloning and expression analysis of cinnamoyl-CoA reductase (CCR) genes in sorghum. PeerJ 4:e2005. 10.7717/peerj.2005 27231650 PMC4878380

[B44] Li L. Cheng X. Lu S. Nakatsubo T. Umezawa T. Chiang V. L. (2005). Clarification of cinnamoyl-coenzyme A reductase catalysis in monolignol biosynthesis of aspen. Plant Cell Physiol. 46, 1073–1082. 10.1093/pcp/pci120 15870094

[B45] Lüderitz T. Grisebach H. (1981). Enzyme synthesis of lignin precursors comparison of cinnamoyl-CoA reductase and cinnamyl alcohol:NADP+ dehydrogenase from spruce (*Picea abies* L.) and soybean (*Glycine max* L.). Eur. J. Biochem. 199, 115–124. 10.1111/j.1432-1033.1981.tb05584.x 7042334

[B46] Ma Q. H. (2007). Characterization of a cinnamoyl-CoA reductase that is associated with stem development in wheat. J. Exp. Bot. 58, 2011–2021. 10.1093/jxb/erm064 17452751

[B47] Ma Q. H. Tian B. (2005). Biochemical characterization of a cinnamoyl-CoA reductase from wheat. Biol. Chem. 386, 553–560. 10.1515/BC.2005.065 16006242

[B48] Miedes E. Vanholme R. Boerjan W. Molina A. (2014). The role of the secondary cell wall in plant resistance to pahtogens. Front. Plant Sci. 5:358. 10.3389/fpls.2014.00358 25161657 PMC4122179

[B49] Moura J. C. M. S. Bonine C. A. V. Vlana J. O. F. Dornelas M. C. Mazzafera P. (2010). Abiotic and biotic stresses and changes in lignin content and composition in plants. J. Int. Plant Biol. 52, 360–376. 10.1111/j.1744-7909.2010.00892.x 20377698

[B50] Nimz H. Ebel J. Grisebach H. (1975). On the structure of lignin from soybean cell suspension cultures. Z. Naturforsch. 30c, 442–444.

[B51] Oerke E. C. (2006). Crop lesses to pests. J. Agric. Sci. 144, 31–43. 10.1017/S0021859605005708

[B52] Pan H. Zhou R. Louie G. V. Mühlemann J. K. Bomati E. K. Bowman M. E.. (2014). Structural studies of cinnamoyl-CoA reductase and cinnamyl-alcohol dehydrogenase, key enzymes of monolignol biosynthesis. Plant Cell 26, 3709–3727. 10.1105/tpc.114.127399 25217505 PMC4213152

[B53] Park H. L. Lee S. W. Jung K. H. Hahn T. R. Cho M. H. (2013). Transcriptomic analysis of UV-induced reveals UV-induced phytoalexins biosynthetic pathways and their regulatory networks in rice. Phytochemistry 96, 57–71. 10.1016/j.phytochem.2013.08.012 24035516

[B54] Park H. L. Yoo Y. Hahn T. R. Bhoo S. H. Lee S. W. Cho M. H. (2014). Antimicrobial activity of UV-induced phenylamides from rice leaves. Molecules 19, 18139–18151. 10.3390/molecules191118139 25383752 PMC6271653

[B55] Pichon M. Courbou I. Beckert M. Boudet A. M. Grima-Pettenati J. (1998). Cloning and characterization of two maize cDNAs encoding cinnamoyl-CoA reductase (CCR) and differential expression of the corresponding genes. Plant Mol. Biol. 38, 671–676. 10.1023/A:1006060101866 9747812

[B56] Piquemal J. Lapierre C. Myton K. C'Connell A. Schuch W. Grima-Pettenati J. (1998). Down-regulation of cinnamoyl-CoA reductase induces significant changes of lignin profiles in transgenic tobacco plants. Plant J. 13, 71–83. 10.1046/j.1365-313X.1998.00014.x

[B57] Rejeb I. B. Pastor V. Mauch-Mani B. (2014). Plant responses to simultaneous biotic and abiotic stress: molecular mechanisms. Plants 3, 458–475. 10.3390/plants3040458 27135514 PMC4844285

[B58] Sarni F. Grand C. Boudet A. M. (1984). Purification and properties of cinnamoyl-CoA reductase and cinnamyl alcohol dehydrogenase from poplar stems (*Populus* X *euramericana*). Eur. J. Biochem. 139, 259–265. 10.1111/j.1432-1033.1984.tb08002.x 6365550

[B59] Satake H. Koyama T. Bahabadi S. E. Matsumoto E. Ono E. Murata J. (2015). Essences in metabolic engineering of lignin biosynthesis. Metabolites 5, 270–290. 10.3390/metabo5020270 25946459 PMC4495373

[B60] Shi R. Sun Y. H. Li Q. Heber S. Sederoff R. Chiang V. L. (2010). Towards a systems approach for lignin biosynthesis in *Populus trichocarpa*: transcript abundance and specificity of the monolignol biosynthetic genes. Plant Cell Physiol. 51, 144–163. 10.1093/pcp/pcp175 19996151

[B61] Srivastava S. Vishwakarma R. K. Arafat Y. A. Gupta S. K. Khan B. M. (2015). Abiotic stress induces changes in cinnamoyl CoA reductase (CCR) protein abundance and lignin deposition in developing seedling of *Leucaena leucocephala*. Physiol. Mol. Biol. Plants 21, 197–205. 10.1007/s12298-015-0289-z 25931776 PMC4411380

[B62] Strange R. N. Scott P. R. (2005). Plant disease: a threat to global food security. Annu. Rev. Phytopathol. 43, 1–34. 10.1146/annurev.phyto.43.113004.133839 16078878

[B63] Stuible H. P. Kombrink E. (2001). Identification of the substrate specificity-conferring amino acid residues of 4-coumarate:coenzyme A ligase allows the rational design of mutant enzymes with new catalytic properties. J. Biol. Chem. 276, 26893–26897. 10.1074/jbc.M100355200 11323416

[B64] Tamasloukht B. Wong Quai Lam M. S. Martinez Y. Tozo K. Barbier O. Jourda C.. (2011). Characterization of a cinnamoyl-CoA reductase 1 (CCR1) mutant in maize: effects on lignification, fibre development, and global gene expression. J. Exp. Bot. 62, 3837–3848. 10.1093/jxb/err077 21493812 PMC3134344

[B65] Tamura K. Stecher G. Peterson D. Filipski A. Kumar S. (2013). MEGA 6: molecular evolutionary genetics analysis version 6.0. Mol. Biol. Evol. 30, 2725–2729. 10.1093/molbev/mst197 24132122 PMC3840312

[B66] Teponno R. B. Kusari S. Spiteller M. (2016). Recent advances in research on lignans and neolignans. Nat. Prod. Rep. 33, 1044–1092. 10.1039/C6NP00021E 27157413

[B67] Thompson J. D. Higgins D. G. Gibson T. J. (1994). CLUSTAL W: improving the sensitivity of progressive multiple sequence alignment through sequence weighting, position-specific gap penalties and weight matrix choice. Nucleic Acids Res. 22, 4673–4680. 10.1093/nar/22.22.4673 7984417 PMC308517

[B68] Vanholme R. Demedts B. Morreel K. Ralph J. Boerjan W. (2010). Lignin biosynthesis and structure. Plant Physiol. 153, 895–905. 10.1104/pp.110.155119 20472751 PMC2899938

[B69] Vinocur B. Altman A. (2005). Recent advances in engineering plant tolerance to abiotic stress: achievements and limitations. Curr. Opin. Biotechnol. 16, 123–132. 10.1016/j.copbio.2005.02.001 15831376

[B70] Xu Z. Zhang D. Hu J. Zhou X. Ye X. Reichel K. L.. (2009). Comparative genome analysis of lignin biosynthesis gene families across the plant kingdom. BMC Informatics 10(Suppl. 11):S3. 10.1186/1471-2105-10-S11-S3 19811687 PMC3226193

