# Supplementary material for: Biochemical and Expression Analyses of the Rice Cinnamoyl-CoA Reductase Gene Family
Source: Front Plant Sci. 2017 Dec 12;8:2099. doi: 10.3389/fpls.2017.02099 (PMC5732984; doi:10.3389/fpls.2017.02099)
Supplement: Supplementary file 1 [file Table1.DOCX]

Supplementary Table 1. Primer sequences of for *OsCCR*s and *At4CL1* cloning, and PCR conditions.

| Gene | Primer sequence ^a^ | Annealing  Temp. (℃) |
| --- | --- | --- |
| *OsCCR1*  (Os02g56460) | 5’-CATATGTCGTCCAATTTTGAGGC-3’  5’-AAGCTTTTATAGGTAAGCACGTTTTG-3’ | 50 |
| *OsCCR5*  (Os01g18120) | 5’-GCTAGCATGGTCACCGGCCGGAGCGA-3’  5’-GAATTCTCACAGGGCTGAACGGCGTG-3’ | 50 |
| *OsCCR17*  (Os09g04050) | 5’-CATATGCCGACTGACGAGACAGC-3’  5’-GAATTCTCACGAGGAAGGGTGGTGGT-3’ | 52 |
| *OsCCR18*  (Os08g17500) | 5’-CATATGGGTGTCGAGAAGACGAC-3’  5’-GAATTCTCACGGCATGGCGGCGGCAG-3’ | 52 |
| *OsCCR19*  (Os09g25150) | 5’-CATATGGCGGCGATGGCGTCGCCG-3’  5’-GAATTCTCAGTTCTCAACAAGAAAGTG-3’ | 52 |
| *OsCCR20*  (Os08g34280) | 5’-CATATGACCGTGATCGACGGCGC-3’  5’-GAATTCTCATGCTCGGATGGCAATCC-3’ | 52 |
| *OsCCR21*  (Os02g08420) | 5’-CATATGGCCGCCGCCGTCGTCTG-3’  5’-GAATTCTCAAAGCTTCGACATCAGCA-3’ | 52 |
| *OsCCR26*  (Os01g74660) | 5’-CATATGGAGGAGGAGCGGCGCGT-3’  5’-AAGCTTTCAAGGAAGGAAGCATTTAT-3’ | 50 |
| *At4CL1*  (At1g51680) | 5’-CATATGGCGCCACAAGAACAAGC-3’  5’-GGATCCTCACAATCCATTTGCTAGTT-3’ | 53 |

^a^ Underline indicate the restriction sites in pET28a(+)
