# Supplementary material for: Biochemical and Expression Analyses of the Rice Cinnamoyl-CoA Reductase Gene Family
Source: Front Plant Sci. 2017 Dec 12;8:2099. doi: 10.3389/fpls.2017.02099 (PMC5732984; doi:10.3389/fpls.2017.02099)
Supplement: Supplementary file 2 [file Table2.DOCX]

Supplementary Table 2. Primer sequences for quantitative real-time PCR analysis.

| Gene | Primer sequence |
| --- | --- |
| *OsCCR17*  (Os09g04050) | 5’-AGCGAGTGGTGTTCACGTCC-3’  5’-CGTCTTCGCGTAGCAGTACC-3’ |
| *OsCCR19*  (Os09g25150) | 5’-CAAGCTCTTCCCCGAGTATCC-3’  5’-GCTGCTTCCGTGGGTTCAT-3’ |
| *OsCCR20*  (Os08g34280) | 5’-GCGTCGTGGCTCGTGAA-3’  5’-CGCGTTCTTCGGGTCATCT-3’ |
| *OsCCR21*  (Os02g08420) | 5’-AGCACCAAGCACATTCTCAAGTAC-3’  5’-GGCGTGGGACTCGTTGAC-3’ |
| *UBQ5*  (Os01g22490) | 5’-ACCACTTCGACCGCCACTACT-3’  5’-ACGCCTAAGCCTGCTGGTT-3’ |
