# Supplementary material for: Biochemical and Expression Analyses of the Rice Cinnamoyl-CoA Reductase Gene Family
Source: Front Plant Sci. 2017 Dec 12;8:2099. doi: 10.3389/fpls.2017.02099 (PMC5732984; doi:10.3389/fpls.2017.02099)
Supplement: Supplementary file 3 [file Table3.DOCX]

Supplementary Table 3. Sequence homologies between the deduced amino acid sequences of OsCCRs and functional CCRs from other plant species. The values show the percentage of amino acid similarities.

|  | **AtCCR1** | **AtCCR2** | **HvCCR** | **LeCCR1** | **LeCCR2** | **LpCCR** | **PtCCR** | **PvCCR1a** | **PvCCR1e** | **PvCCR2a** | **SbcCR1** | **SbCCR2-1** | **SbCCR2-2** | **TaCCR1** | **TaCCR2** | **ZmCCR1** | **ZmCCR2** |
| --- | --- | --- | --- | --- | --- | --- | --- | --- | --- | --- | --- | --- | --- | --- | --- | --- | --- |
| **OsCCR1** | 65 | 64 | 67 | 69 | 69 | 67 | 66 | 65 | 65 | 61 | 63 | 67 | 61 | 65 | 64 | 63 | 65 |
| **OsCCR2** | 34 | 34 | 36 | 35 | 35 | 36 | 33 | 36 | 35 | 30 | 36 | 35 | 29 | 36 | 34 | 35 | 35 |
| **OsCCR3** | 36 | 45 | 37 | 37 | 37 | 38 | 36 | 36 | 36 | 33 | 36 | 36 | 33 | 36 | 34 | 35 | 36 |
| **OsCCR4** | 65 | 65 | 65 | 70 | 70 | 66 | 68 | 62 | 62 | 62 | 61 | 65 | 61 | 65 | 63 | 60 | 64 |
| **OsCCR5** | 66 | 66 | 66 | 71 | 70 | 67 | 69 | 63 | 63 | 61 | 62 | 65 | 61 | 66 | 63 | 62 | 65 |
| **OsCCR6** | 54 | 53 | 54 | 56 | 55 | 55 | 53 | 53 | 54 | 50 | 51 | 52 | 49 | 54 | 49 | 51 | 52 |
| **OsCCR7** | 49 | 49 | 49 | 50 | 50 | 49 | 48 | 46 | 47 | 44 | 45 | 48 | 45 | 48 | 46 | 45 | 48 |
| **OsCCR8** | 49 | 50 | 49 | 52 | 51 | 50 | 49 | 47 | 48 | 44 | 46 | 49 | 45 | 49 | 47 | 46 | 49 |
| **OsCCR10** | 64 | 64 | 66 | 67 | 67 | 65 | 65 | 64 | 65 | 61 | 63 | 65 | 61 | 65 | 63 | 62 | 64 |
| **OsCCR11** | 62 | 63 | 65 | 67 | 66 | 65 | 65 | 63 | 63 | 58 | 61 | 64 | 57 | 64 | 62 | 61 | 63 |
| **OsCCR12** | 65 | 65 | 67 | 69 | 69 | 67 | 67 | 65 | 65 | 61 | 63 | 67 | 62 | 66 | 64 | 63 | 65 |
| **OsCCR13** | 62 | 62 | 65 | 66 | 66 | 65 | 65 | 65 | 65 | 59 | 64 | 65 | 57 | 65 | 63 | 64 | 64 |
| **OsCCR14** | 45 | 44 | 44 | 46 | 45 | 44 | 45 | 43 | 43 | 43 | 43 | 44 | 44 | 44 | 43 | 43 | 44 |
| **OsCCR15** | 49 | 47 | 49 | 49 | 49 | 49 | 48 | 47 | 47 | 45 | 47 | 48 | 46 | 48 | 46 | 47 | 47 |
| **OsCCR16** | 45 | 45 | 47 | 47 | 45 | 46 | 45 | 44 | 45 | 42 | 45 | 45 | 42 | 47 | 43 | 45 | 44 |
| **OsCCR17** | 72 | 70 | 73 | 73 | 75 | 74 | 70 | 74 | 73 | 69 | 72 | 89 | 68 | 72 | 85 | 72 | 87 |
| **OsCCR18** | 68 | 66 | 69 | 68 | 69 | 69 | 66 | 71 | 70 | 65 | 68 | 75 | 64 | 69 | 71 | 67 | 74 |
| **OsCCR19** | 75 | 72 | 91 | 75 | 75 | 90 | 72 | 86 | 86 | 65 | 83 | 75 | 65 | 90 | 74 | 82 | 73 |
| **OsCCR20** | 77 | 75 | 87 | 76 | 76 | 86 | 74 | 91 | 92 | 64 | 89 | 74 | 63 | 85 | 73 | 86 | 74 |
| **OsCCR21** | 66 | 65 | 67 | 67 | 69 | 67 | 66 | 64 | 64 | 88 | 62 | 70 | 91 | 66 | 70 | 62 | 69 |
| **OsCCR22** | 54 | 53 | 55 | 56 | 55 | 56 | 53 | 53 | 53 | 51 | 52 | 53 | 50 | 55 | 51 | 52 | 53 |
| **OsCCR23** | 34 | 34 | 36 | 35 | 35 | 37 | 33 | 36 | 36 | 30 | 36 | 37 | 30 | 36 | 35 | 35 | 36 |
| **OsCCR24** | 64 | 66 | 65 | 69 | 69 | 65 | 66 | 62 | 62 | 58 | 61 | 64 | 58 | 64 | 62 | 62 | 63 |
| **OsCCR25** | 47 | 49 | 50 | 50 | 51 | 50 | 48 | 47 | 48 | 48 | 46 | 48 | 49 | 50 | 46 | 45 | 48 |
| **OsCCR27** | 12 | 11 | 12 | 12 | 12 | 12 | 12 | 11 | 11 | 11 | 11 | 12 | 11 | 12 | 11 | 11 | 13 |
| **OsCCR28** | 21 | 23 | 22 | 23 | 23 | 22 | 22 | 21 | 21 | 19 | 20 | 21 | 20 | 22 | 20 | 21 | 20 |
| **OsCCR29** | 34 | 34 | 34 | 36 | 36 | 35 | 35 | 33 | 33 | 34 | 32 | 34 | 33 | 35 | 33 | 31 | 35 |
| **OsCCR30** | 28 | 27 | 29 | 30 | 30 | 29 | 28 | 27 | 27 | 26 | 27 | 27 | 26 | 29 | 27 | 27 | 27 |
| **OsCCR31** | 20 | 21 | 20 | 21 | 21 | 20 | 20 | 19 | 19 | 19 | 18 | 20 | 19 | 20 | 19 | 18 | 20 |
| **OsCCR32** | 37 | 36 | 37 | 38 | 39 | 37 | 38 | 35 | 35 | 35 | 34 | 36 | 35 | 37 | 35 | 34 | 36 |
| **OsCCR33** | 34 | 34 | 34 | 35 | 35 | 34 | 35 | 32 | 32 | 34 | 31 | 35 | 34 | 34 | 34 | 31 | 35 |
| **OsCCR34** | 18 | 18 | 19 | 19 | 18 | 18 | 18 | 18 | 18 | 15 | 18 | 17 | 15 | 18 | 17 | 18 | 17 |
| **OsCCR35** | 19 | 20 | 19 | 20 | 20 | 19 | 19 | 18 | 18 | 18 | 18 | 19 | 19 | 19 | 18 | 18 | 19 |
