# Supplementary material for: Biochemical and Expression Analyses of the Rice Cinnamoyl-CoA Reductase Gene Family
Source: Front Plant Sci. 2017 Dec 12;8:2099. doi: 10.3389/fpls.2017.02099 (PMC5732984; doi:10.3389/fpls.2017.02099)
Supplement: Supplementary file 4 [file Table4.DOCX]

Supplementary Table 4. Enzyme activity assay of the recombinant OsCCRs with *p*-coumaroyl-, feruloyl-, and sinapoyl-CoAs^a^.

|  | **OsCCR1** | **OsCCR5** | **OsCCR17** | **OsCCR19** | **OsCCR20** | **OsCCR21** | **OsCCR26** |
| --- | --- | --- | --- | --- | --- | --- | --- |
| ***p*-Coumaroyl-CoA** | –^b^ | – | +^c^ | + | + | + | – |
| **Feruloyl-CoA** | – | – | + | + | + | + | – |
| **Sinapoyl-CoA** | – | – | + | + | + | + | – |

^a^ CCR activity was measured according to the method of Lüderitz and Grisebach (1981). Hydroxycinnamoyl-CoA substrate (30 μM) and 5 μg of purified recombinant OsCCR protein were used in the activity assay.

^b^ Minus signs indicate no detectable enzyme activity.

^c^ Plus signs indicate the enzyme has the catalytic activity to the examined substrate.
