# Supplementary material for: Biochemical and Expression Analyses of the Rice Cinnamoyl-CoA Reductase Gene Family
Source: Front Plant Sci. 2017 Dec 12;8:2099. doi: 10.3389/fpls.2017.02099 (PMC5732984; doi:10.3389/fpls.2017.02099)
Supplement: Supplementary file 5 [file Table5.DOCX]

Supplementary Table 5. Lignin compositions of stems of the Dongjin rice cultivar used in this study^a^.

| Monolignol | H-unit | G-unit | S-unit | Total |
| --- | --- | --- | --- | --- |
| μmol/g DW | 4.63 ± 0.28 | 103.55 ± 1.75 | 59.40 ± 3.99 | 167.59 ± 2.06 |

^a^ Monolignol analysis of rice stems was carried out according to the method described by Foster et al. (*J. Vis. Exp.* 37, e1745, 2010). Stem samples were collected from ten week-old adult rice plants and used for analysis of lignin compositions. Lignin analysis was performed on three triplicated biological samples and each sample was analyzed three-times for technical replicate. The results represent mean ± standard deviation. DW; dry weight.
