# Supplementary material for: Biochemical and Expression Analyses of the Rice Cinnamoyl-CoA Reductase Gene Family
Source: Front Plant Sci. 2017 Dec 12;8:2099. doi: 10.3389/fpls.2017.02099 (PMC5732984; doi:10.3389/fpls.2017.02099)
Supplement: Supplementary file 6 [file Image1.PDF]

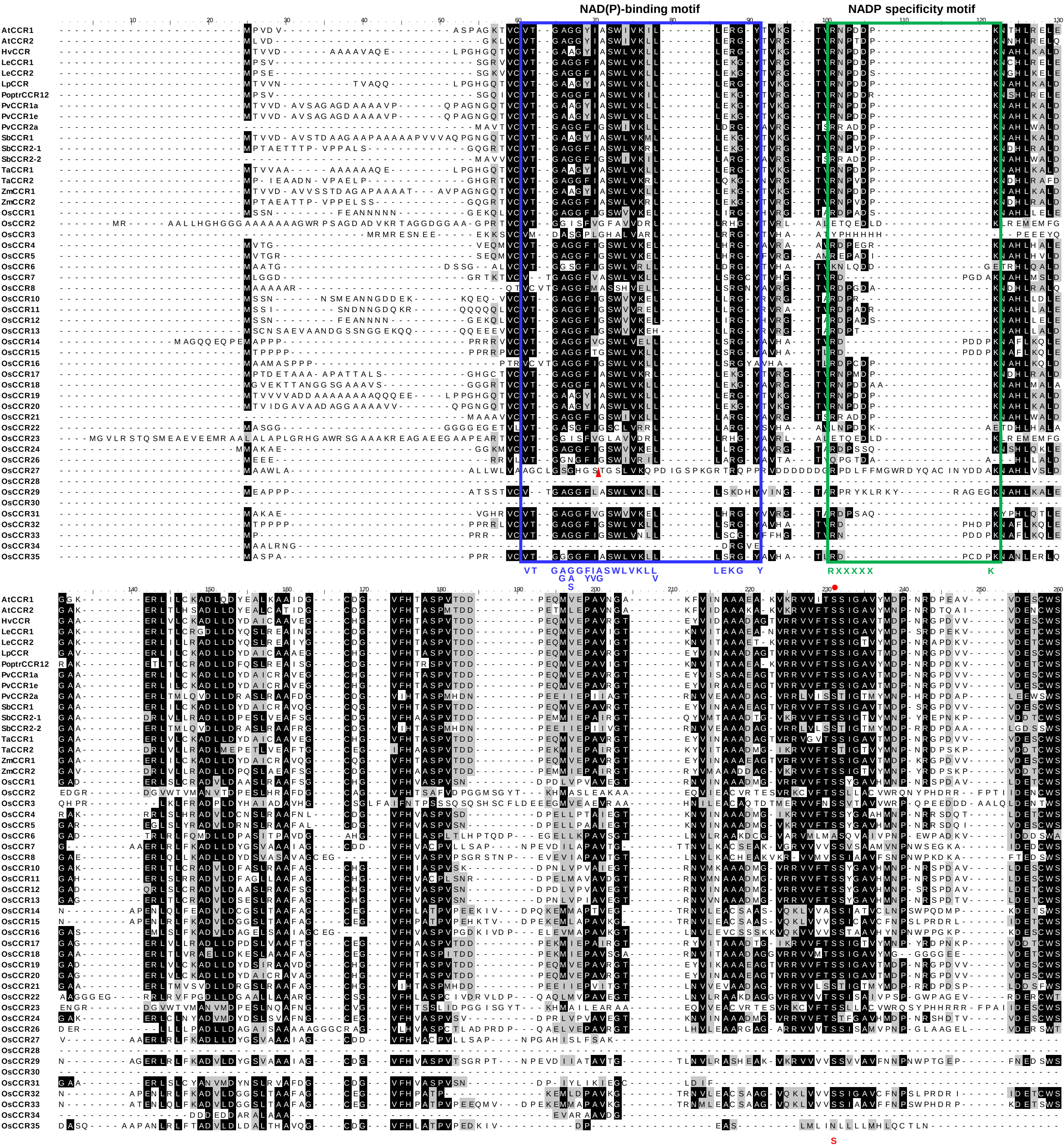

NAD(P)-binding motif
NADP specificity motif
VT GAGGFIASWLVKLL LEKG Y
GA YVG V
S
RXXXXX K
S

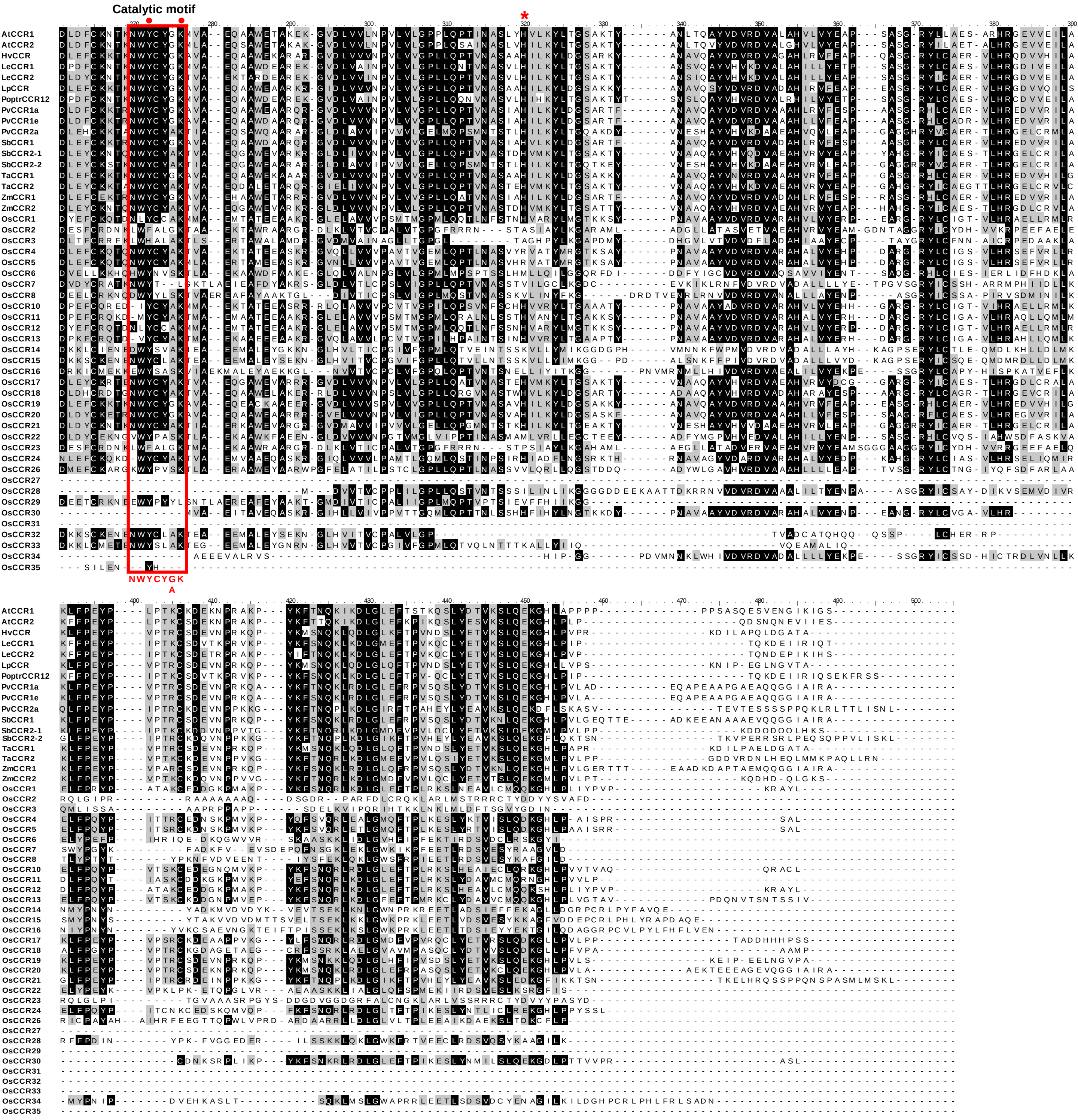

Supplementary Figure 1. Multiple sequence alignments of the deduced amino acid sequences of OsCCRs with CCRs from other plant species. The amino acid sequences were alingned using Clustal-W. The shaded amino acids denoted identical or similar amino acids. The blue and green box indicate NAD(P)-binding and NADP specificity motif, respectively. The red box indicates catalytic motif. The converved amino aid residues are indicated below the aliments. A red asterisk indicates H208 in PtoCCR. The catalytic triad Ser-Tyr-Lys is indicated by red circles. A red triangle in the N-terminus of OsCCR27 indicates the cleavage site of secretory signal peptide. *A. thaliana* CCRs (AtCCR1, AAG46037; AtCCR2, AAG53687); *H. vulgare* CCR (HvCCR, AAN71760); *L. esculentum* CCRs (LeCCR1, AAY41879.1; LeCCR2, AAT41880.1); *L. perenne* CCR (LpCCR, AAG09817.1); *P. trichocarpa* CCR (PoptrCCR, CAA12276.1); *P. virgatum* CCRs (PvCCR1a, GQ450297; PvCCR1e, GQ450301; PvCCR2a, GQ450302); *S. bicolor* CCRs (SbCCR1, XP002445566.1; SbCCR2-1, EER98579.1; SbCCR2-2, EES04640.1); *T. aestivum* CCRs (TaCCR1, ABE01883; TaCCR2, AY771357); *Z. mays* CCRs (ZmCCR1, CAA74071; ZmCCR2, NP_001005715).
