# Supplementary material for: Biochemical and Expression Analyses of the Rice Cinnamoyl-CoA Reductase Gene Family
Source: Front Plant Sci. 2017 Dec 12;8:2099. doi: 10.3389/fpls.2017.02099 (PMC5732984; doi:10.3389/fpls.2017.02099)
Supplement: Supplementary file 7 [file Image2.PDF]

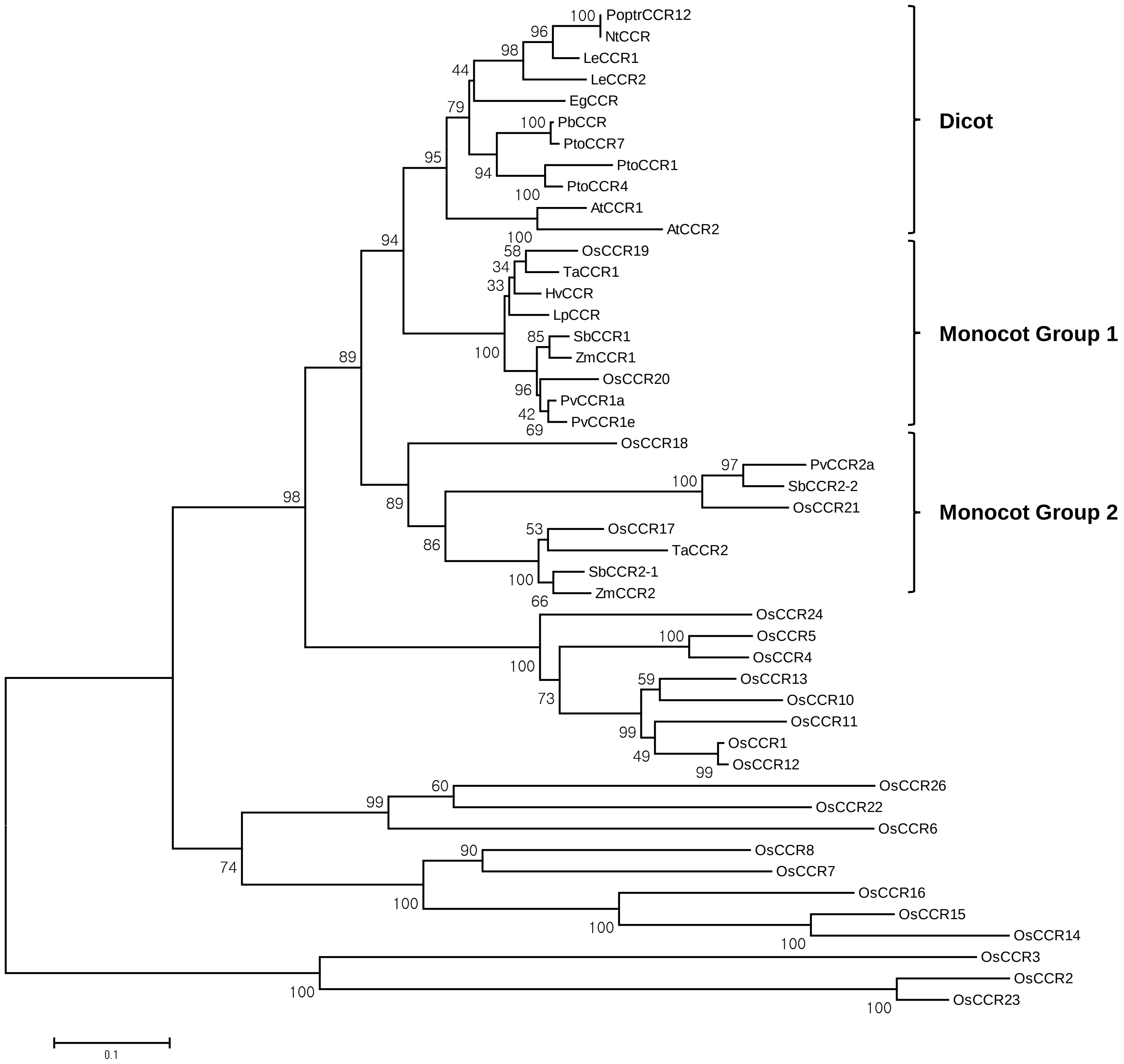

Supplementary Figure 2. Phylogenetic analysis of OsCCRs and 17 characterized CCRs from other plant species. The the neighbor-joining tree was built using MEGA6. Dicot and monocot CCRs are separately grouped. Monocot CCRs are divided into two groups, Group 1 and 2. Monocot Group 1 includes the functional CCRs involved in the developmental lignification and Group 2 contains the defense-related CCRs. *A. thaliana* CCRs (AtCCR1, AAG46037; AtCCR2, AAG53687); *H. vulgare* CCR (HvCCR, AAN71760); *L. esculentum* CCRs (LeCCR1, AAY41879.1; LeCCR2, AAT41880.1); *L. perenne* CCR (LpCCR, AAG09817.1); *P. trichocarpa* CCR (PoptrCCR, CAA12276.1); *P. virgatum* CCRs (PvCCR1a, GQ450297; PvCCR1e, GQ450301; PvCCR2a, GQ450302); *S. bicolor* CCRs (SbCCR1, XP002445566.1; SbCCR2-1, EER98579.1; SbCCR2-2, EES04640.1); *T. aestivum* CCRs (TaCCR1, ABE01883; TaCCR2, AY771357); *Z. mays* CCRs (ZmCCR1, CAA74071; ZmCCR2, NP_001005715); *E. gunnii CCR* (EgCCR, X79566); *P. balsamifera* CCR (PbCCR, AJ295838); *N. tabacum* CCR (NtCCR, A47101); *P. tomentosa* CCRs (PtoCCR1, KP281597; PtoCCR4, KP281599; PtoCCR7, KF145198).
