# Supplementary material for: Biochemical and Expression Analyses of the Rice Cinnamoyl-CoA Reductase Gene Family
Source: Front Plant Sci. 2017 Dec 12;8:2099. doi: 10.3389/fpls.2017.02099 (PMC5732984; doi:10.3389/fpls.2017.02099)
Supplement: Supplementary file 8 [file Image3.PDF]

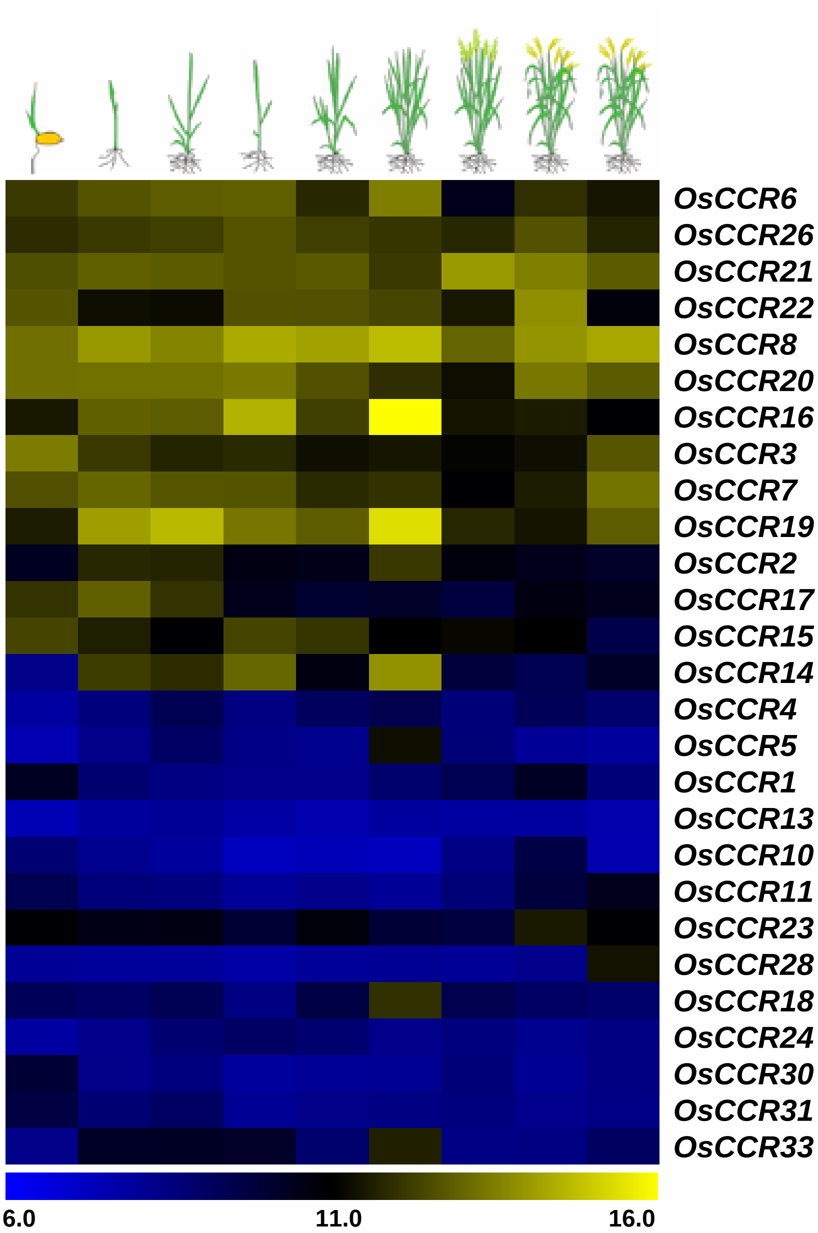

Supplementary Figure 3. Expression patterns of *OsCCR*s in different developmental stages of rice plants. The developmental stages examined were seed germination, seedling, tillering, stem elongation, booting, heading, flowering, milk and dough stages. Meta-expression analysis of different rice developmental stages was performed with microarray data obtained from the Genevestigator plant biology database. Heatmaps were generated using Multi Experiment Viewer program (http://www.tm4.org/mev.html).
