# Supplementary material for: Biochemical and Expression Analyses of the Rice Cinnamoyl-CoA Reductase Gene Family
Source: Front Plant Sci. 2017 Dec 12;8:2099. doi: 10.3389/fpls.2017.02099 (PMC5732984; doi:10.3389/fpls.2017.02099)
Supplement: Supplementary file 9 [file Image4.PDF]

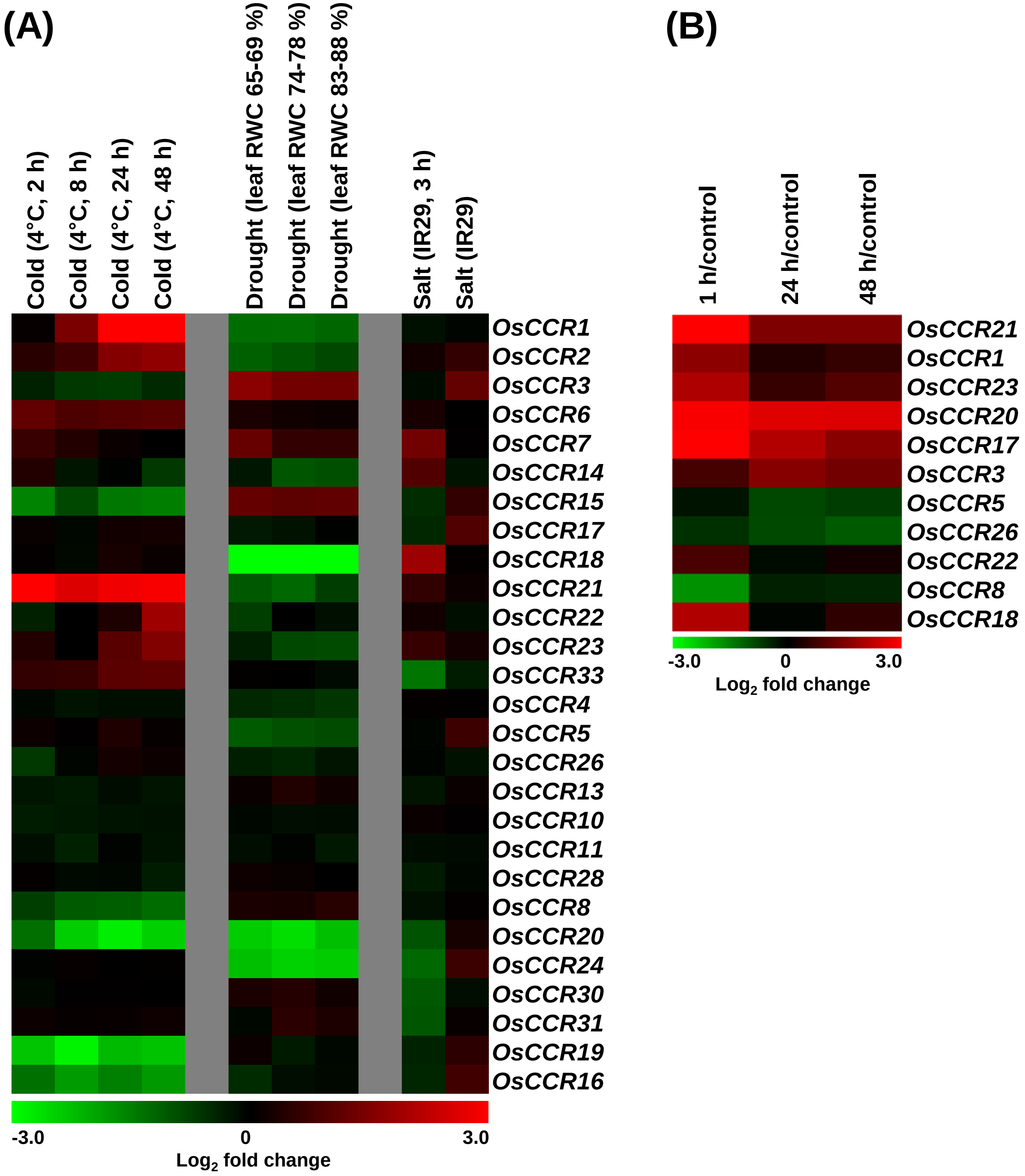

Supplementary Figure 4. *In silico* microarray analysis of *OsCCR*s expression in response to abiotic stress. (A) The expression patterns of *OsCCR*s in cold-, drought- and salt-treated rice samples. Misroarray data of in cold-, drought- and salt-treated rice samples were obtained from the Genevestigator plant biology database. (B) The expression patterns of *OsCCR*s in UV-treated rice leaves. Microarray analysis of rice leaves 1, 24 and 48 h after UV treatment were analyzed previously (Park et al., 2013). The color scale represents the $\log_2$ fold changes of gene expression in response to abiotic stress conditions, cold, drought, salt and UV treatment. Heatmaps were generated using Multi Experiment Viewer program (http://www.tm4.org/mev.html).
