# Supplementary material for: Biochemical and Expression Analyses of the Rice Cinnamoyl-CoA Reductase Gene Family
Source: Front Plant Sci. 2017 Dec 12;8:2099. doi: 10.3389/fpls.2017.02099 (PMC5732984; doi:10.3389/fpls.2017.02099)
Supplement: Supplementary file 10 [file Image5.PDF]

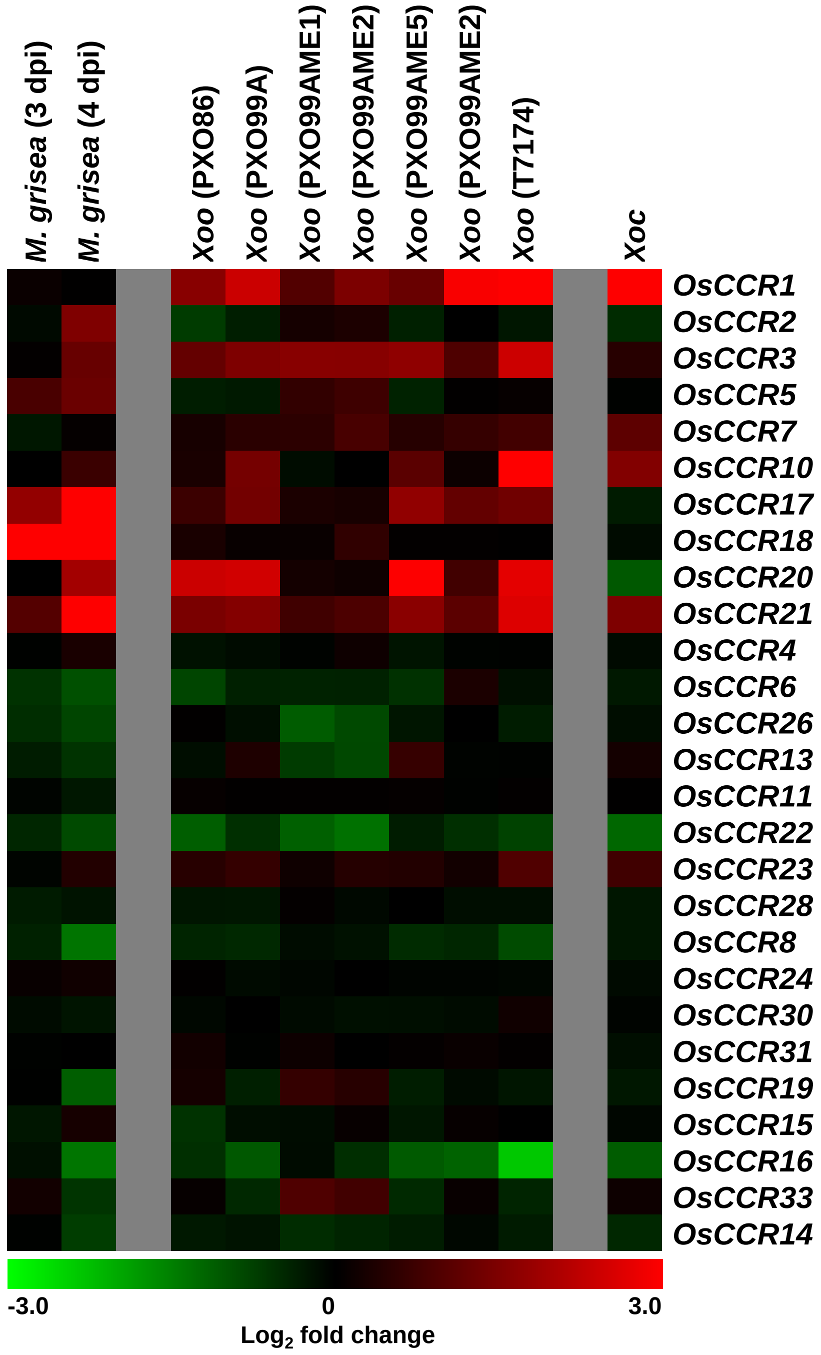

Supplementary Figure 5. *In silico* microarray analysis of *OsCCR* gene expression in response to biotic stress. The expression patterns of *OsCCR*s were analyzed by 3 and 4 days after infection of *M. grisea*. The expression patterns of *OsCCR*s in rice infected with different pathogenic lines of *Xoo*. The color scale represents the $\log_2$ fold changes of gene expression in response to biotic stresses, such *M. grisea*, *X. oryzae* pv. *oryzae* (*Xoo*) and *X. oryzae* pv. *oryzicolar* (*Xoc*) infections.
